# Concomitant human papillomavirus (HPV) vaccination and screening for elimination of HPV and cervical cancer

**DOI:** 10.1038/s41467-024-47909-x

**Published:** 2024-05-01

**Authors:** Laila Sara Arroyo Mühr, Andrea Gini, Emel Yilmaz, Sadaf S. Hassan, Camilla Lagheden, Emilie Hultin, Ainhoa Garcia Serrano, Agustin E. Ure, Helena Andersson, Roxana Merino, K. Miriam Elfström, Iacopo Baussano, Joakim Dillner

**Affiliations:** 1 Center for Cervical Cancer Elimination, Department of Clinical Science, Intervention and Technology (CLINTEC), Karolinska Institutet and Karolinska University Hospital, F56, Stockholm, Sweden; 2 International Agency for Research on Cancer (IARC/WHO), Early Detection, Prevention and Infections Branch, Lyon, France

**Keywords:** Population screening, Viral infection, Human papilloma virus

## Abstract

HPV vaccination with concomitant HPV-based screening of young women has been proposed for faster cervical cancer elimination. We describe the baseline results of a population-based trial of this strategy to reduce the incidence of HPV. All 89,547 women born 1994-1999 and resident in the capital region of Sweden were personally invited to concomitant HPV vaccination and HPV screening with 26,125 women (29.2%) enrolled between 2021-05-03 and 2022-12-31. Baseline HPV genotyping of cervical samples from the study participants finds, compared to pre-vaccination prevalences, a strong decline of HPV16 and 18 in birth cohorts previously offered vaccination, some decline for cross-protected HPV types but no decline for HPV types not targeted by vaccines. Our dynamic transmission modelling predicts that the trial could reduce the incidence of high-risk HPV infections among the 1994-1998 cohorts by 62-64% in 3 years. Baseline results are prevalences of HPV infection, validated transmission model projections, and power estimates for evaluating HPV incidence reductions at follow-up (+/−0.1% with 99.9% confidence). In conclusion, concomitant HPV vaccination and HPV screening appears to be a realistic option for faster cervical cancer elimination. Clinicaltrials.gov identifier: NCT04910802; EudraCT number: 2020-001169-34.

## Abstract (web summary)

Here the authors report baseline results of a population-based trial testing concomitant human papillomavirus (HPV) vaccination and HPV-based screening of young women in Sweden and, using a transmission model, suggest that this approach may reduce high-risk HPV infections.

## Introduction

Vaccination against human papillomavirus (HPV) and organized cervical HPV screening are the most powerful tools to eliminate cervical cancer^1^. HPV vaccination will eventually eliminate vaccine-targeted types of HPV from the population by herd immunity when vaccinated children become adults^2^. Similarly, HPV screening will eventually eliminate cervical cancer when the entire population has been screened for HPV^1^.

The WHO global cervical cancer elimination strategy includes HPV vaccination of 90% of girls by the age of 15, 70% of women screened using a high-performance test by the age of 35, and again by the age of 45, and 90% of women with cervical disease treated^1^. Several countries have already reached these goals, but population-based declines of cervical cancer are as yet limited. For example, in Sweden, 89.8% of girls ages 10–12 in 2021 have been vaccinated with at least one dose (National Public Health Agency website https://www.folkhalsomyndigheten.se/publikationer-och-material/publikationsarkiv/b/barnvaccinationsprogrammet-i-sverige-2022-arsrapport/ accessed on 20231226), 82% of women ages 23–70 had been screened according to recommendations (National Cervical Screening Registry website www.nkcx.se, accessed on 20231226), and 96% of women with high-grade cervical lesions or worse (HSIL+) had been biopsied within a year (www.nkcx.se, accessed on 20231226) but the incidence of cervical cancer is still 11/100,000 women/year (National Board of Health and Welfare website www.socialstyrelsen.se, accessed at 20231226). There is therefore interest in design and piloting of strategies that could result in an even faster elimination of HPV and cervical cancer. Screening has the advantage that it has no time component (once the whole population has been screened, the full effect of the screening program is instantly seen). However, because there are still new HPV infections being spread, the screening may need to be repeated. A major bottleneck for achieving a rapid elimination is therefore that there is a substantial spread of HPV among women in age groups that are not targeted for vaccination^3^.

We and others have previously demonstrated that: 1) extending HPV vaccination strategies to birth cohorts that are still propagating HPV infections at a reproductive rate >1 should result in a faster elimination of incident HPV infections in the whole population^3^ and that 2) women who receive concomitant HPV vaccination and HPV screening will have a near-complete and long-lasting protection against cervical cancer^4–6^.

In the Swedish population, there is little propagation of HPV among women above 30 years of age and women 23 years of age or younger will have been targeted by high coverage school-based HPV vaccination programs^3^, resulting in that it is the age groups between 23-30 years of age that are still propagating HPV in Sweden. HPV vaccination in schools started in 2012 for girls and in 2020 for boys. School-based vaccination has resulted in high coverage (between 80-90%). At the start of the program the girls aged 13–18 were offered a catch-up vaccination, that had suboptimal coverage (~55%). The vaccine used during 2012–2019 was Gardasil4, with a switch to Gardasil9 in 2020. The rationale for the trial described in this report is to investigate if a trial with population-based invitations to HPV vaccination (with Gardasil9) and HPV screening to all women aged 23-29 in the population will result in a reduced incidence of HPV infections in the entire population.

While the advantage of faster HPV elimination by this strategy is easily understood, implementation of this type of strategy has been hampered by the fact that the strategy needs new logistics (feasibility) and more large-scale input data for validating models and estimating the impact of the intervention (power of trials).

We therefore launched a nationwide population-based study offering all women in the country who were born 1994–1999 (aged 23–29) concomitant HPV vaccination and HPV-screening. The trial was performed in 2 phases, starting first in 2021 with the capital region of Sweden (Stockholm region, ~25% of the population of Sweden) and then a second phase with enrollment in the rest of the country from autumn 2022 until the end of 2024. The follow-up visit with new HPV screening to measure if the intervention has affected HPV prevalences and incidences will be 3 years after enrollment. Two different implementation strategies were used: (1) “Campaign” using vaccination sites, where vaccinated women are screened using HPV self-sampling and (2) “Organized screening” where the women in the population who are due for screening are invited for concomitant screening and vaccination at a specified time and place - with vaccination given by the midwife that previously would take only the screening sample. The reasoning was that the campaign strategy would probably be faster and less expensive. However, using the established infrastructure of the organized screening program could possibly reach a higher attendance and have better long-term stability.

The trial is currently in a nationwide enrollment phase, targeting all >350,000 women born 1994–1999 in Sweden. The trial is formally a scientific clinical trial, registered at clinicaltrials.gov and with permissions from the Swedish Ethical Review Authority and Medical Products Agency. However, the launch of the trial was decided on by the Swedish parliament and by all the regional healthcare providers of Sweden. The major financial support is a specific line item in the national budget of Sweden, decided on by the Swedish parliament (see “Acknowledgement” section).

The rationale of starting the trial first in the capital region was to gain experiences that could be useful for the nationwide implementation. The present report describes the baseline results of enrolling for HPV vaccination, the results of the HPV genotyping tests obtained until 2022-12-31 and provides the 3-year model predictions on HPV incidence that the trial will be evaluated against.

## Results

### Vaccination and HPV screening

**Fig. 1:**
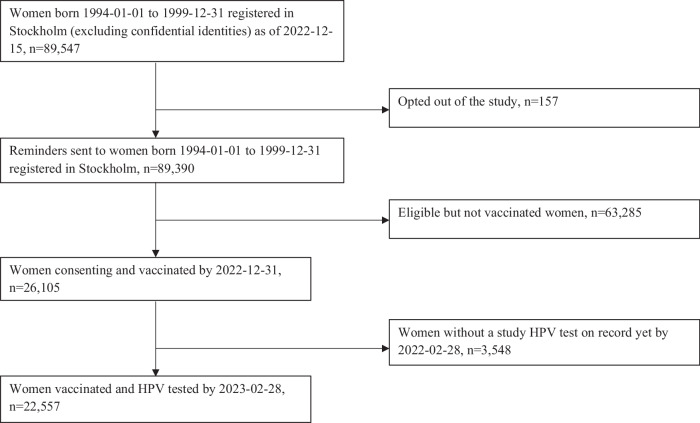
Study flow-chart. Vaccinated and HPV-tested women were included in the study.

The target population of women born between 1994-1999 and residing in the greater Stockholm County was extracted from the population registry (that includes contact information, domicile, and the registered sex) and found to encompass 89,547 women. All received personal invitations to participate in the trial. One hundred and fifty-seven women (0.18%) declined participation, leaving a target group of 89,390 women (Fig. 1).

There were 26,105 women (29.2% of the total target population) who consented, were vaccinated with Gardasil9, and offered HPV testing (either by cervical sampling by midwives or as self-sampling) between 2021-05-03 and 2022-12-31 (Table 1). For the present paper, we included all HPV tests submitted and analyzed by 28 February 2023 (2 months after the last vaccination date, allowing for reasonable delays in taking the sample, posting, and analyzing it). In this analysis, only one sample per individual is considered (the first sample taken after vaccination). By 28 February 2023, 22,557/26,105 women (86.4%) had an HPV test on file taken at or after the vaccination (Table 1). Among these, most HPV tests (13,460/22,557, 59.7%) were based on self-sampling. There were 8667 midwife-taken samples (38.4%) from the organized program and a few clinical samples (430 samples (1.91%)). The exact reason for taking these clinical samples is not known, but it could be, for instance, that a woman had a scheduled appointment with a private gynecologist and did not take the self-sample because of the gynecologist-taken sample.

**Table 1:** Type-specific HPV prevalences at baseline enrollment in the population-based trial of concomitant HPV vaccination and HPV screening

Birth year	Number of women in population	Prior Vaccination offered	*n* (%) of those women vaccinated in trial	*n* (%) of those women HPV tested	HPV 16	HPV18	HPV45	HPV 33/58	HPV 31	HPV 52	Low oncogenic HPV (35/39/51/56/59/66/68)	Other HPV^a^	HPV neg	*n* (%) with valid HPV test
					*n* (%)	*n* (%)	*n* (%)	*n* (%)	*n* (%)	*n* (%)			*n* (%)	
1994	18,436	Catch-up	5687	4941	122	26	118	191	106	117	475	136	3609	4900
			(30.85)	(86.88)	(2.47)	(0.53)	(2.39)	(3.87)	(2.15)	(2.37)	(9.61)	(2.75)	(73.04)	(99.17)
1995	16,761	Catch-up	5120	4428	98	24	148	170	98	126	456	92	3175	4387
			(30.55)	(86.48)	(2.21)	(0.54)	(3.34)	(3.84)	(2.21)	(2.85)	(10.30)	(2.08)	(71.70)	(99.07)
1996	15,619	Catch-up	4788	4176	72	18	118	138	74	131	421	161	3012	4145
			(30.65)	(87.22)	(1.72)	(0.43)	(2.83)	(3.30)	(1.77)	(3.14)	(10.08)	(3.86)	(72.13)	(99.26)
1997	13,971	Catch-up	4108	3465	73	26	93	143	65	97	372	164	2405	3438
			(29.40)	(84.35)	(2.11)	(0.75)	(2.68)	(4.13)	(1.88)	(2.80)	(10.74)	(4.73)	(69.41)	(99.22)
1998	12,802	Catch-up	4020	3419	73	16	67	125	41	77	270	425	2303	3997
			(31.40)	(85.05)	(2.14)	(0.47)	(1.96)	(3.66)	(1.20)	(2.25)	(7.90)	(12.43)	(67.36)	(99.43)
1999	11,801	School-based	2382	2128	15	1	34	92	22	81	246	39	1584	2368
			(20.18)	(89.34)	(0.70)	(0.05)	(1.60)	(4.32)	(1.03)	(3.81)	(11.56)	(1.83)	(74.44)	(99.41)
Total	89,390	–	26,105	22,557	453	111	578	859	406	629	2240	1017	16,088	22,381
			(29.20)	(86.41)	(2.01)	(0.49)	(2.56)	(3.81)	(1.80)	(2.79)	(9.93)	(4.51)	(71.32)	(99.22)

^a^“Other HPV” corresponds to the results obtained as a pool from the Cobas platform (HPV types 31/33/35/39/45/51/52/56/58/59/66/68) that could not be retested in the BD platform for extended genotyping (i.e., the exact genotype is unknown). Comparison can also be made to population-based HPV prevalences in Sweden in these age groups at the time when no HPV vaccination had been offered. This has been previously published and was 30% for any HPV positivity, 6.1% for HPV 16, and 2.4% for HPV 18^8^.

In 2022, the organized screening program switched from partial (HPV16/18/Other) (Cobas 4800) to extended HPV genotyping (BD Onclarity) for HPV screening. Therefore, samples positive for “other HPV” in partial genotyping were re-analyzed with extended genotyping. Among 14,377 samples analyzed only with partial genotyping, 4146 (28.84%) were positive for “other HPV” and these were re-analyzed with extended genotyping. For 3129/4146 (75.47%) of such specimens, results were obtained for extended genotyping, but 1017 samples (24.53%) could not be re-analyzed (sample not biobanked or low quantity of sample material left).

About 28% of samples were HPV-positive (Table 1). However, most of the HPV positivity was for the low oncogenicity HPV types that are not targeted by the nonavalent HPV vaccine. The three most oncogenic HPV types - HPV 16, HPV 18, and HPV 45 - were present in 2.0%, 0.5%, and 2.6% of the samples, respectively (Table 1). Valid HPV results were ascertained in 99.22% of samples (Table 1).

The prevalence of the major HPV types targeted by the first-generation vaccine (HPV 16 and 18) was low among the women who had been offered organized, school-based HPV vaccination that achieved high coverage (born 1999)^7^. In total, 15 HPV 16 infections and 1 HPV 18 infection (prevalence of 0.70% and 0.05%) were detected in the 1999 cohort (Table 1). An effect of vaccination on HPV prevalences was also seen in women offered catch-up vaccination (born 1994–1998, moderate coverage) (Table 1). Population-based pre-vaccination HPV prevalences in Sweden in these age groups has been previously published and was 30% for any HPV positivity, 6.1% for HPV 16, and 2.4% for HPV 18^8^. The prevalences of HPV types not targeted by the vaccine (and not having known cross-protection) seemed stable among the different birth cohorts (Table 1).

### Predicted effects of the trial

**Fig. 2:**
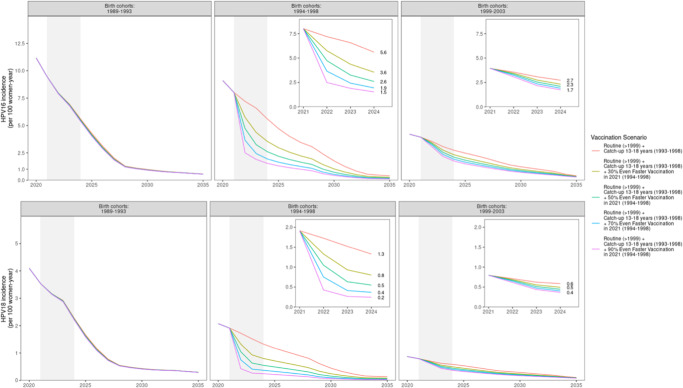
Projected HPV16 and HPV 18 incidence over time (year 2020–2035), by HPV vaccination scenario and birth cohort. Projected HPV 16 and HPV 18 incidence over time for different vaccination scenarios and birth cohorts.

The projected incidence over time of HPV 16 (upper panels) and HPV 18 (lower panels) as a function of possible attendance rates in the trial are shown in Fig. 2 (with an extension in Supplementary Tables S1 and S2), starting with the observed 30% attendance rate in Stockholm County as of 2022-12-31 (present study) and with predicted gains if attendance can be increased (with time), or if enrollment is more successful in other counties.

As expected, the largest gains in reduction of HPV 16/18 incidence are found in the birth cohorts targeted by the trial who had previously been targeted only by catch-up vaccination with suboptimal coverage (born 1994–1998). For example, this age group had an HPV 16 incidence of 9.1 (per 100 women-year) in 2020 which, with the trial attendance reported in this paper (30%), will decline to 3.6 in 2024, as compared to the decline expected if no trial had been launched (from 9.1 to 5.6). As shown in Fig. 2 insets, increasing the trial coverage will further reduce incidence. There were negligible gains in the birth cohorts that did not have any organized vaccination before and were not targeted by the trial (born 1989–1993). For example, the HPV 16 incidence decreased in this age group from 11.2 (per 100 women-year) in 2020 to 1.2 in 2028 and further to 0.5 in 2035 – but with the same decline seen regardless of the current trial. There are some gains in the age group targeted by high coverage school-based vaccination (born 1999-2003) where only the girls born 1999 are targeted by the trial (as comparison group). The HPV 16 incidence is 4.2 (per 100 women-year) in 2020 and this will decline to 2.3 in 2024 (assuming a 30% trial attendance), as compared to the 2.7 incidence predicted in 2024 if no trial had been launched). The timing between the enrollment test and the trial follow-up test (3 years) is displayed as a shaded area. As can be seen, the full protection of the trial is not realized immediately as it takes some time before secondary protection (herd immunity) develops. Similar patterns are predicted for HPV 18 (Fig. 2).

## Discussion

The present study has provided data on the feasibility of implementing concomitant HPV vaccination and HPV screening aiming to achieve faster elimination of HPV and eventually also of cervical cancer. Furthermore, it provided the large-scale input data needed to enable a model-based prediction of the effects of implementing the strategy.

Key feasibility results include that it was simple to use a dual strategy using both vaccination centers that also do screening as well as using screening sites that also do vaccinations. Linking self-sampling kits and their subsequent analysis results to the vaccinated study participants was readily doable, which has implications for other efforts seeking to combine vaccination and screening. Most of the attendance came from the campaign-based strategy. However, it remains to be demonstrated whether the sequential strategy of using nested vaccinations within the routine organized screening program may eventually result in higher attendance.

Key model-based predictions include that a much faster decline of HPV incidences is expected in the same birth cohorts as targeted by the trial. The predicted, limited effect among older cohorts may seem surprising, as it is known that HPV has a very low reproductive rate among older women^3^. Young women, ages 17–18, have historically been the most active in spreading HPV; however, women with ages 12–23 years of age have nowadays very low HPV prevalences as they have been vaccinated with high coverage in schools. The decline of HPV is therefore quite steep already. It was also somewhat surprising that quite a large effect was predicted at the current 30% population coverage of the trial, possibly a result of that the number of infectious individuals in the population is rapidly declining which could make it easier to break transmission chains.

Finally, the result of HPV testing among the women born 1999 deserves to be noted, as there was only one woman positive for HPV 18 out of more than 2000 women tested, implying that already at the start of the trial, HPV 18 is about to become extinct. This HPV type used to be a very common virus that caused >15% of all cervical cancers^9^. If the trial can accelerate the elimination of this virus, it would indeed be a major gain.

Strengths of the study include that the design is innovative, large-scale, and population-based with personal invitations, the (re)use of the real-life infrastructure for screening and vaccination, well characterized dynamic transmission models and the use of the same extended genotyping platform as used for routine screening, providing generalizability.

Weaknesses include that it was, for ethical reasons, not possible to include a placebo group and that HPV incidences are expected to decline anyhow (regardless of the intervention), necessitating evaluation of the follow-up of HPV prevalences by comparing with the model-based predicted occurrence of HPV at the calendar time for the follow-up testing (after three years). Prevalence of non-vaccine types remained unchanged across the birth cohorts included in the study (with varying previous exposure to vaccine) implying that exposure is similar and any changes seen in vaccine-type prevalence result from the HPV vaccinations. Another weakness is that we modeled only the 2 major HPV types (HPV16/18), because these were the only oncogenic types previously vaccinated against.

Although the strategy with combined HPV vaccination and HPV screening for faster cervical cancer elimination is well-described in the literature^3–5^ and the effects of multi-age-cohort vaccination are elaborated on in numerous modeling studies^10,11^, the present implementation trial is to our knowledge the first real-life nationwide and population-based implementation of the strategy. WHO member states have unanimously agreed to pursue cervical cancer elimination as a priority^12^. As there are large advances in implementation of key prevention tools (HPV vaccination and HPV screening) but more limited gains in cervical cancer prevention, research investigating possible strategies to achieve a faster elimination are in great demand. Apart from trial results from studies like ours, key health outcomes like cancer cases/deaths, efficiency outcomes like numbers needed to treat and cost-effectiveness would be important for considerations on how best to optimize elimination strategies.

Many studies have investigated the cost-effectiveness of different cervical cancer prevention strategies. The results from randomized clinical trials clearly document that HPV vaccination of adult women works well, provided that women are HPV-screened at the time of HPV vaccination and found to be HPV negative^13,14^. By contrast, the effect of vaccinating older women without considering baseline HPV status is limited^13,14^. Somewhat surprisingly, most studies of cost-effectiveness of vaccinating adult women focused on vaccination without concomitant HPV testing. The present trial does not consider cost-effectiveness of various policies. The trial evaluates the effect of the intervention, that is it evaluates whether concomitant HPV vaccination and HPV screening will affect HPV incidences in the population. In conclusion, the present study shows that the logistics of concomitant HPV vaccination and HPV screening are feasible and has modeled the predicted effects of this strategy. The concept and data may be useful in the continued work towards the elimination of HPV and cervical cancer.

## Methods

### Study population

All women born 1994-1999 and resident in the Stockholm County during 2021–2022 were eligible to be included. Women were identified through the Total Population Registry of Sweden (at the Swedish Tax Agency), which includes the registered sex of all residents. The registry is essentially complete, but a few women may have protected identity (witness protection, etc.). The women with protected identity were not invited, but all women born 1994–1999 were welcome to participate if they had heard about the trial and showed up at a vaccination site. This was rather common, for example, for women resident in other parts of Sweden or students from other countries who were not formally resident in the area. Males were not eligible for vaccination. For men with a cervix (transgender men) we set up a separate vaccination site. As the protocol specified “women”, we were not able to include transgender men in the trial but we offered them the same HPV vaccinations and HPV tests as we offered to women. Vaccinations of transgender males or non-resident females are not included in this report, neither in the denominator nor in the numerator.

Informed consent was required to participate. Exclusion criteria were non-consent and contraindications to vaccination (current reported pregnancy or known history of severe allergic reaction or hypersensitivity to any of the components of the HPV vaccine) as well as self-reported total hysterectomy. Any suspected unexpected serious adverse reactions as well as any potential immune mediated disease are duly reported to health authorities as obliged by and following the adequate procedures detailed by the Swedish Medical Products Agency.

### Recruitment strategies

All women in the target population (*n* = 89,547, as registered in 2022-12-15) received population-based mobile phone push messages, reminders, and physical letters with invitations to the elimination campaign. The messages included “Anyone born 1994-1999 can book free vaccination and screening”, “Protect everyone – Get the jab against HPV”, and “Tell your friends”. Furthermore, television (news) and social media posts by prestigious and well-known partners (The Swedish Cancer Society and the patient association www.Gyncancer.se) collaborated in spreading the message.

#### Campaign

In the campaign strategy, most of the participation was at the major COVID-19 vaccination centers of the Karolinska University Hospital, that for several months had two waiting lines (one for COVID vaccination and one for HPV vaccination). The initial strategy for screening was to collect the lists of consenting, vaccinated subjects and send a self-sampling kit to their home address. The vaccination sites deemed that labeling of tubes was not compatible with their high throughput flow of vaccinations. Subsequently, we developed a system where the tube had a unique identity that the woman herself could link to her identity and from that point the vaccination sites distributed the self-sampling kit after vaccination with instructions to the woman on how to link the kit to her identity. The campaign strategy also offered women to book a time for vaccination and screening at a maternity care center (71 such centers exist all over the Stockholm region, meaning that the need for travel was minimized). However, these sites had limited excess capacity.

#### Organized cervical screening program

The organized cervical screening program invites all women ages 23-25 to midwife-based cervical screening with specified time and place in the invitation. As of 2021, individual invitations to midwife-based screening are no longer issued to women aged 26 or older, as these women are instead offered self-sampling for HPV by mailing self-sampling kits to their home address. The standard invitation letter to screening was replaced with a standard invitation letter to screening and vaccination. The letter includes instructions on how to rebook the appointment if the time or location are inconvenient. The midwives at the 71 maternity care centers contracted by the program to perform routine screening were educated in vaccinology by an on-line course. The centers could choose to either take the cervical sample as usual or to distribute a self-sampling kit (to save time in case of limited capacity). Most centers opted for cervical sampling by the midwife.

### Vaccination and self-sampling

For this trial, the dosing schedule approved by the Swedish Medical Products Agency was 2 doses of Gardasil9^©^ given with 3 years interval. The vaccinator first confirmed existence of informed consent and assessed that vaccination was not contraindicated (e.g., ongoing pregnancy) and then vaccinated the participant with Gardasil9. Cervical sampling was completed using liquid-based cytology (Cytobrush and ThinPrep vials) or self-sampling. The self-sampling kit included instructions, the swab, a tube with media to collect the vaginal sample, a plastic zip bag containing an absorbent pad (for the sample tube to be placed inside to avoid spills and leaks when sending to the laboratory for analysis), a QR code for the woman herself to link the kit to her identity, and a pre-paid return envelope for sample submission.

### HPV analysis and HPV results management

HPV analysis was performed at Karolinska University Hospital (the laboratory of the organized screening program), using the same laboratory and HPV screening platform as used by the organized screening program. This was initially the Cobas 4800 platform (Roche Diagnostics) but was in 2022, following a new tender for the screening program, switched to the BD Onclarity™ HPV Assay that provides an extended genotyping. Samples that had tested positive for “other HPV” on the Cobas platform were re-analyzed using the BD Onclarity™ HPV Assay to ensure the same level of genotyping detail in the entire database. Both assays were fully proficient in the HPVLabNet proficiency testing (limit of detection at least 10 IU/µl for HPV16/18 and 100 IU/µl for the other HPV types)^15^.

Results were both available to the women (who could log in to the database online), the screening program and associated health care, and exported to the Swedish National Cervical Screening Registry (http://nkcx.se). HPV-positive women were followed up according to the established national program of care for cervical cancer prevention (available online at https://kunskapsbanken.cancercentrum.se/globalassets/vara-uppdrag/prevention-tidig-upptackt/gynekologisk-cellprovskontroll/vardprogram/nationellt-vardprogram-cervixcancerprevention.pdf, accessed on August 25th, 2023).

### Ethics

All women provided informed consent to participate in the study and filled out a health declaration prior to vaccination. Both the health declaration and consent were initially collected using paper forms, but we developed an open-source vaccination and screening platform for electronic and real-time collection of the data (stockholm.hpvelimination.se). A QR code to enter this platform was provided with the mobile phone push messages, in the physical letters, as well as posted in the waiting rooms of the maternity care and vaccination centers, such that the women would have time to read and provide their consent and health declaration before their appointment. The trial is approved by the Swedish Ethical Review Authority (Decision number 2020-07145) and the Swedish Medical Products Agency (Decision number 5.1-2021-8496) and registered with the EU Clinical Trials Register (EudraCT 2020-001169-34) and Clinicaltrials.gov (NCT04910802).

### Statistical analysis

#### Participation and HPV prevalence

The primary outcome of interest was prevalence of HPV infection. Women who provided informed consent and were vaccinated between 2021-05-03 and 2022-12-31 were included in this analysis of trial participation rates and type-specific HPV prevalences at baseline enrollment. HPV tests submitted for analysis until 2023-02-28 were included in the baseline calculations. Participation until December 2022 was estimated as the proportion of women who were vaccinated in the trial among the target population of women born 1994-1999. The proportion of women with a concomitant HPV test on record within the follow-up timeframe was determined among those participating. HPV type-specific prevalences were reported as absolute numbers and proportions by birth-cohort. Precision was estimated using exact confidence intervals for proportions.

The primary outcome of the trial will be evaluated after the next study visit, 3 years after enrollment. The pre-defined statistical analysis that will be used is a time trends analysis where possible abrupt changes trends over time (as expected when birth cohorts targeted by school-based vaccinations are entering the screening program) will be analyzed using joinpoint analysis of population-based HPV screening data. In addition, the observed HPV incidences at follow-up will be compared to the expected incidences, estimated as described in this paper.

#### Dynamic transmission modeling

To predict the impact of the study on HPV 16 and HPV 18 incidences in the entire population, we adapted our established dynamic, population-based, single-type HPV transmission model, previously fitted to the Swedish population^16,17^.

Briefly, a total of 100,000 sets of parameter values were generated by independently sampling a uniform distribution for each parameter within a pre-specified value range to calibrate the original model. Each set of values generated a model-based type- and age-specific prevalence curve. For each HPV type, we then calculated the binomial log-likelihood to assess the fit of each model’s output to the above-mentioned age-specific prevalence. Finally, we adjusted the current version of the model to the HPV type- and age-specific HPV prevalences among unvaccinated women in Sweden^8^ by adapting the sexual activity of the simulated population and the probability of transmission (only for HPV 18). For the present article only the results of the final model are presented. We selected the ten model-generated curves that fitted the observed data best and validated the models as described below. We simulated HPV incidence (per 100 women-year) over the period 2020 to 2035 under five scenarios (no trial and 4 different possible population participation rates in the trial). In the first scenario (a), we used the actual data on coverage in school-based vaccination at age 11–12 years (ranging from 80 to 90%)^18^ for women born in 1999 and later and the actual data on coverage from the organized catch-up that had targeted women aged 13-18 years (coverage around 55%)^18^ for women born from 1993 to 1998. Then, we simulated the population participation in the trial in 2021 for women born from 1994 to 1998 assuming, respectively, 30% (b), 50% (c), 70% (d), or 90% (e) population participation. Of note, all the scenarios also assume that the current policies of school-based vaccination at age 12 will continue (gender-neutral school vaccination from 2020 onwards). Furthermore, we assumed that both previously Gardasil4-vaccinated and previously unvaccinated women would be enrolled into the trial with similar probabilities. For each scenario, we reported the mean values across each set of 10 projections as a summary measure of the effectiveness of HPV vaccination. Outcomes were computed by HPV type and groups of birth cohorts (1989–1993; 1994–1998; and 1999–2003).

#### Technical model description and validation

The model describes an open population with an age of entrance of 10 years and a maximum age of 80 years. People entering the population are stratified in two Classes of Sexual Activity, (CSAs): 15% were assigned in the high CSA; and 85% in the low. Sex, age, and CSA determines rates of partner acquisition and only partnerships between opposite sex were implemented. Partner acquisition rates were derived from sexual behavior survey data which can be subjected to bias. To adjust for possible bias in assortative patterns, we inserted adjustment parameters in the age and sex-specific assortativeness.

Although the model can simulate any high-risk HPV type^6^, we focused on HPV 16 and 18 as these are the types that are most carcinogenic and cause most cancers. These types are directly targeted by both quadrivalent, bivalent and nonavalent vaccines. Therefore, HPV 16 and HPV 18 are the most relevant types to capture the impact of concomitant HPV vaccine and screening.

The modeled HPV types are assumed to be transmitted independently, governed by type-specific natural history parameters. The natural history parameters include parameters related to the probability of transmission, duration of infection and natural immunity (Supplementary Fig. 1).

HPV vaccination can be inserted into the model at any given time, any given sex, age, and CSA with specific vaccination coverages.

The model was calibrated to country-specific observed data, as described^16,17^. The process followed the method of Van de Velde et al. ^19^. A total of 100,000 sets of parameter values were generated by independent sampling. A uniform distribution within a pre-specified range of values was used for each parameter^20^. We generated a model-based age-specific curve of prevalence for each HPV type using each set of sampled values. We calculated the binomial log-likelihood to compare the model-based age- and HPV type-specific curves of prevalence with the observed outcomes and we selected the 10 model-generated curves that fittest best. Although this classical approach is less efficient than applying a Markov Chain Monte Carlo calibration^21^, the accuracy and validity of the final model parameters are the same.

The calibration initially used the partner acquisition rates from a nation-wide population-based survey^22^, HPV prevalence data was obtained from the voluntary Chlamydia trachomatis screening program^23^. Results of the original calibration and uncertainty of the input parameters are reported in Baussano et al. ^16^. However, as pre-vaccine HPV prevalence data from population-based HPV screening became available in Sweden^8^, our model was adapted to the population-based HPV prevalences (Supplementary Fig. 2). In the adaptation (i) 35% of the simulated individuals were assigned to the high CSA; and (ii) we reduced the transmission probability of HPV 18 by 25%.

The adapted model was validated replicating HPV 16 and HPV 18 prevalences among the women who were enrolled to the trial in this paper (Supplementary Fig. 3). We simulated the entire Swedish population. We inserted organized catch-up vaccination in the model (at age 13-18 years) in women born in 1993-1998 (coverage ~55%) and school-based vaccination at age 11-12 years for women born from 1999 onwards (coverage ranging from 80% to 90%). Gender neutral vaccination was simulated from 2021 onwards. Swedish age-, year-, and birth cohort-specific vaccine coverage data from the National Public Health Agency website statistics was used^18^.

### Reporting summary

Further information on research design is available in the Nature Portfolio Reporting Summary linked to this article.

### Supplementary information

Supplementary Information

Peer Review File

Reporting Summary

## Data Availability

The data generated in this study have been deposited in the Swedish National Cervical Screening Registry database under accession code 2020-001169-34 [www.nkcx.se]. The data are available under restricted access for research use. Access can be obtained by applying to the registry as described on www.nkcx.se (an Institutional Review Board approval must be obtained and a Data Use Agreement must be completed, no other restrictions apply). The raw individual data are protected and are not available due to data privacy laws. The processed data are available at www.nkcx.se. The data generated in this study are provided in the Supplementary Information.

## Code availability

The code used in this analysis can be found at https://gitlab.com/iarc-miarc/analysis/concomitant-hpv. The model is one element of the METHIS (ModElling Tools for HPV Infection-related cancers) project, which is making publicly available a set of open-source models on a platform at the webpage https://iarc-miarc.gitlab.io/methis/methis.website.
