# Supplementary material for: Concomitant human papillomavirus (HPV) vaccination and screening for elimination of HPV and cervical cancer
Source: Nat Commun. 2024 May 1;15:3679. doi: 10.1038/s41467-024-47909-x (PMC11063066; doi:10.1038/s41467-024-47909-x)
Supplement: Supplementary file 1 — Supplementary Information [file 41467_2024_47909_MOESM1_ESM.pdf]

Concomitant Human Papillomavirus (HPV) Vaccination and Screening for Faster Elimination of HPV and Cervical Cancer

SUPPLEMENTARY MATERIAL

Table of contents

Figure S.1 **Page 2**
HPV type transmission model

Figure S.2 **Page 3**
Model-predicted and observed age-specific prevalence of HPV 16 and 18 in Sweden.

Figure S.3 **Page 4**
Model validation based on replication of the observed age specific HPV 16 and HPV 18 prevalence among vaccinated women in Sweden**.**

Table S.1 **Page 5**
Projected HPV 16 incidence over time (year 2020 to 2024, with 80% uncertainty intervals (UI), by HPV vaccination scenario and birth cohort

Table S.2 **Page 6**
Projected HPV 18 incidence over time (year 2020 to 2024, with 80% uncertainty intervals (UI), by possible population participation in trial and birth cohort

Study protocol **Page 7**

Clinicaltrials.gov identifier: NCT04910802.

Figure S1. HPV type transmission model.

The dynamics of the infection transmission accounts for calendar time, women's age, and time elapsed since infection (or infection duration). Note that the dimensions of the arrows connecting the different compartments are not proportional to the rates of transition.

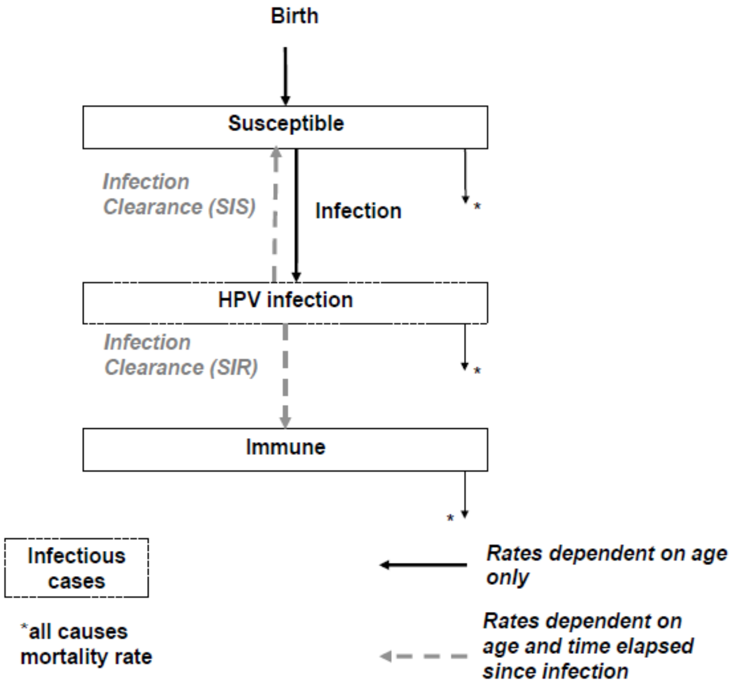

Figure S2. Model-predicted and observed age-specific prevalence of HPV 16 and 18 in Sweden.

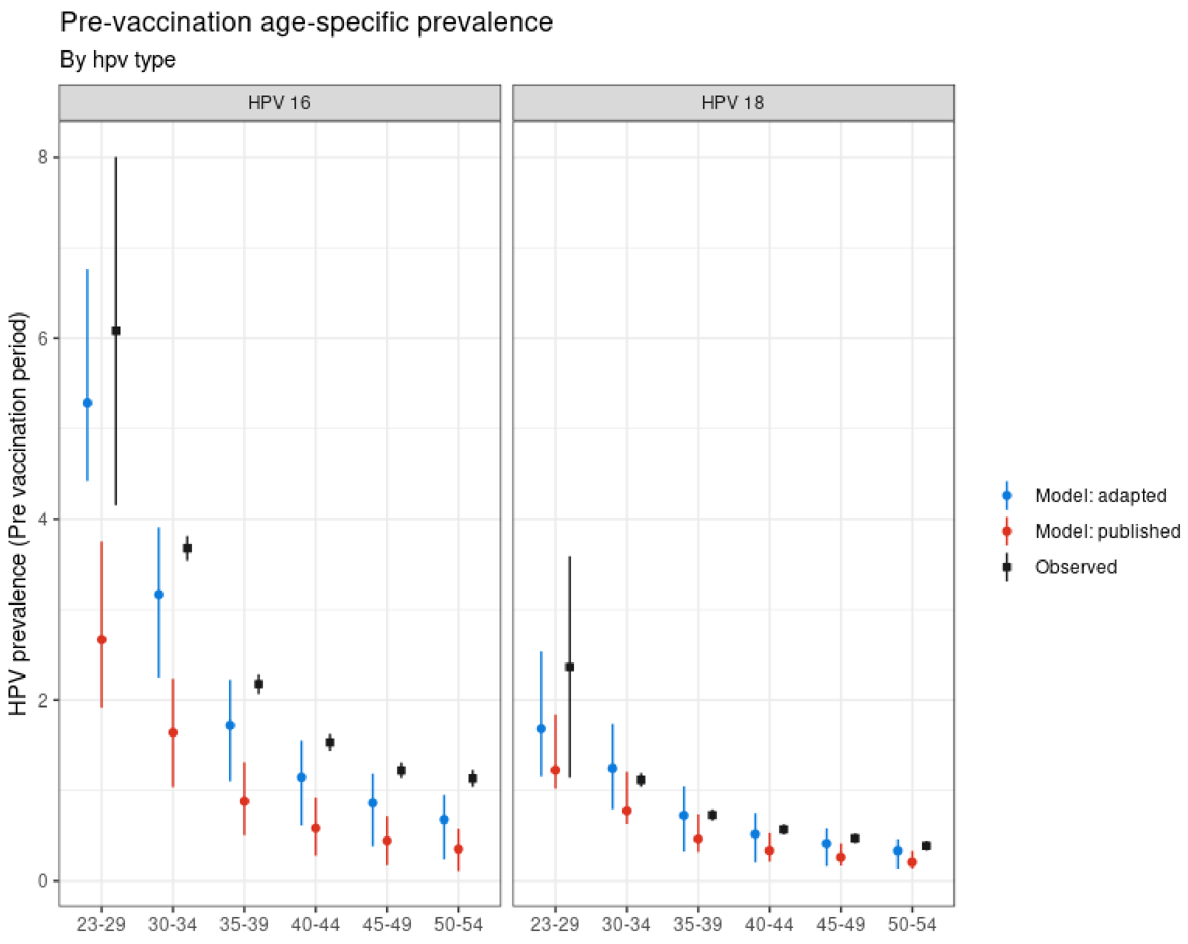

Note: Modelled point values represent the mean value of the 10 best fitting parameters under two assumptions: original published model; and adapted model used for this study. Black square points represent observed values.

Figure S3. Model validation based on replication of the observed age specific HPV 16 and HPV 18 prevalence among vaccinated women in Sweden**.**

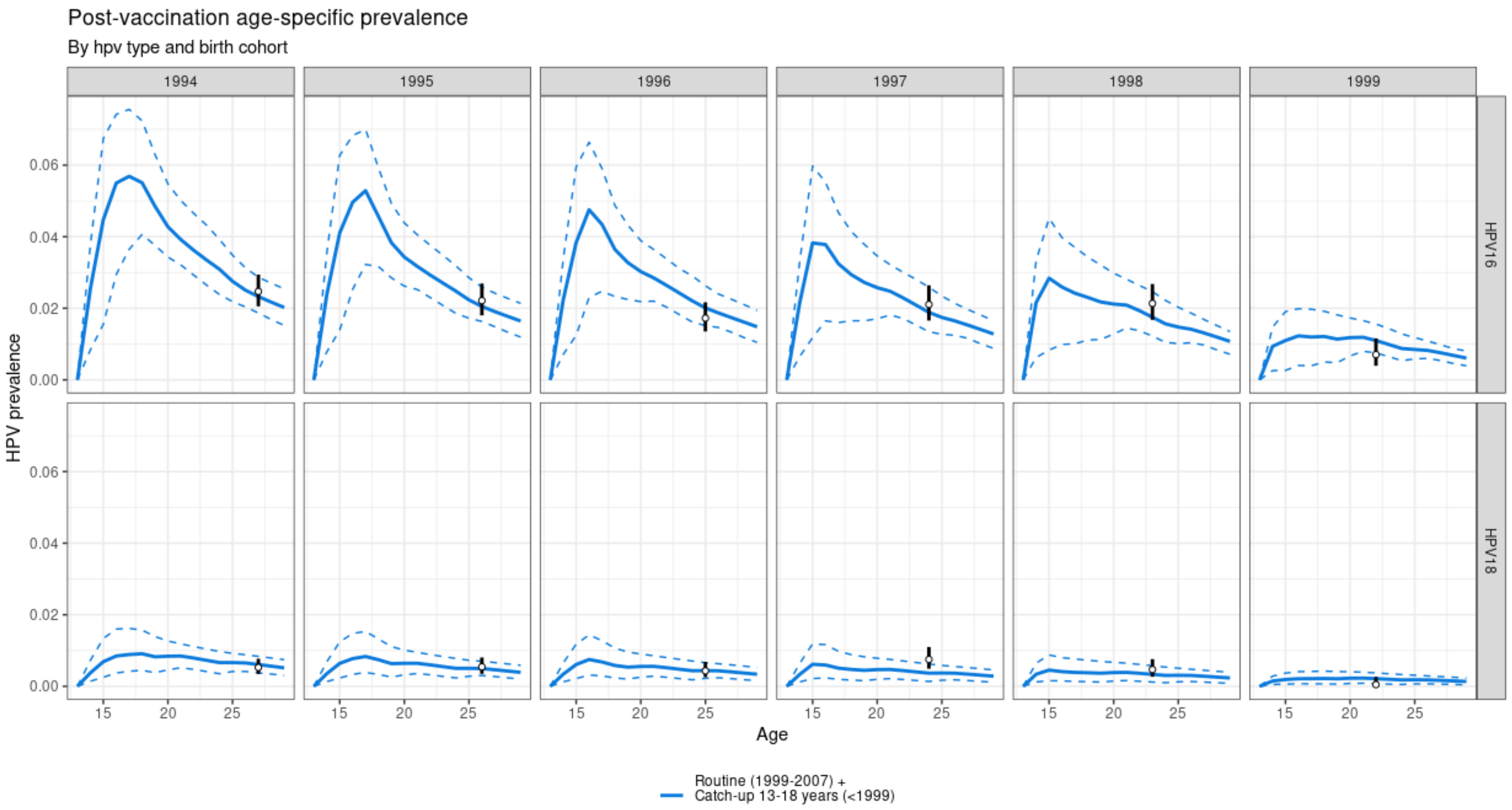

Notes: Black hollow dots represent the HPV 16 and HPV 18 prevalence observed for each birth cohort included in the population-based trial (Table 1; in the model replication we assumed that samples were taken in 2021 for all birth cohorts); the vertical black lines represent the corresponding 95% confidence intervals (computed assuming a binomial distribution).

Table S1. Projected HPV 16 incidence over time (year 2020 to 2024, with 80% uncertainty intervals (UI), by HPV vaccination scenario and birth cohort

**HPV vaccination scenario**	**Calendar year**	**HPV incidence (80% UI) by groups of birth cohorts**		
		**1989-1993**	**1994-1998**	**1999-2003**
No concomitant HPV vaccination and screening	2020	11.2 (6.4-15.2)	9.1 (6.6-14.1)	4.2 (0.7-6.6)
	2021	9.4 (6-13.1)	8 (6-11.7)	3.9 (1.8-5.9)
	2022	8 (5.7-12.3)	7.2 (5.4-9.6)	3.6 (1.9-5.2)
	2023	6.9 (5.1-10.4)	6.6 (5.3-10.8)	3.1 (1.5-4.6)
	2024	5.6 (1.9-7.9)	5.6 (3.7-7.8)	2.7 (1-4.1)
30% Concomitant HPV vaccination and screening	2020	11.2 (6.4-15.2)	9.1 (6.6-14.1)	4.2 (0.7-6.6)
	2021	9.4 (6-13.1)	8 (6-11.7)	3.9 (1.8-5.9)
	2022	8 (5.7-12.3)	5.7 (4.1-7.5)	3.4 (1.9-4.9)
	2023	6.9 (5.1-10.3)	4.4 (3.1-6.5)	2.7 (1.4-4)
	2024	5.5 (1.9-7.8)	3.6 (2.7-5.6)	2.3 (1-3.6)
50% Concomitant HPV vaccination and screening	2020	11.2 (6.4-15.2)	9.1 (6.6-14.1)	4.2 (0.7-6.6)
	2021	9.4 (6-13.1)	8 (6-11.7)	3.9 (1.8-5.9)
	2022	7.9 (5.7-12.3)	4.7 (3.5-6.1)	3.3 (1.8-4.8)
	2023	6.9 (5.1-10.3)	3.3 (2-4.7)	2.5 (1.3-4)
	2024	5.5 (1.9-7.7)	2.6 (1.7-3.8)	2.1 (1-3.4)
70% Concomitant HPV vaccination and screening	2020	11.2 (6.4-15.2)	9.1 (6.6-14.1)	4.2 (0.7-6.6)
	2021	9.4 (6-13.1)	8 (6-11.7)	3.9 (1.8-5.9)
	2022	7.9 (5.7-12.3)	3.7 (2.5-5.1)	3.2 (1.8-4.7)
	2023	6.8 (5.1-10.2)	2.4 (1.3-3.7)	2.4 (1.1-3.9)
	2024	5.4 (1.8-7.7)	1.9 (1.1-2.9)	1.9 (0.9-3.4)
90% Concomitant HPV vaccination and screening	2020	11.2 (6.4-15.2)	9.1 (6.6-14.1)	4.2 (0.7-6.6)
	2021	9.4 (6-13.1)	8 (6-11.7)	3.9 (1.8-5.9)
	2022	7.9 (5.7-12.2)	2.5 (1.5-3.8)	3.1 (1.7-4.7)
	2023	6.8 (5.1-10.2)	1.9 (1-2.9)	2.2 (0.8-3.9)
	2024	5.4 (1.8-7.6)	1.5 (0.8-2.2)	1.7 (0.8-3.4)

Note: 80% UI, 80% Uncertainty intervals.

Table S2. Projected HPV 18 incidence over time (year 2020 to 2024, with 80% uncertainty intervals (UI), by possible population participation in trial and birth cohort

HPV vaccination scenario	Calendar year	HPV incidence (80% UI) by groups of birth cohorts		
		1989-1993	1994-1998	1999-2003
No concomitant HPV vaccination and screening	2020	4.1 (2.8-5.5)	2.1 (1-3)	0.9 (0.3-1.5)
	2021	3.5 (2.5-4.6)	1.9 (1-2.6)	0.8 (0.3-1.4)
	2022	3.2 (2.3-4.3)	1.7 (1.2-2.2)	0.7 (0.3-1.2)
	2023	2.9 (2-4)	1.5 (1.1-1.9)	0.6 (0.2-1.1)
	2024	2.3 (0.9-3.5)	1.3 (1-1.9)	0.6 (0.2-1)
30% Concomitant HPV vaccination and screening	2020	4.1 (2.8-5.5)	2.1 (1-3)	0.9 (0.3-1.5)
	2021	3.5 (2.5-4.6)	1.9 (1-2.6)	0.8 (0.3-1.4)
	2022	3.2 (2.3-4.3)	1.3 (0.9-1.7)	0.7 (0.3-1.2)
	2023	2.9 (2-4)	0.9 (0.7-1.3)	0.6 (0.2-1)
	2024	2.2 (0.9-3.5)	0.8 (0.5-1.2)	0.5 (0.2-0.9)
50% Concomitant HPV vaccination and screening	2020	4.1 (2.8-5.5)	2.1 (1-3)	0.9 (0.3-1.5)
	2021	3.5 (2.5-4.6)	1.9 (1-2.6)	0.8 (0.3-1.4)
	2022	3.2 (2.3-4.3)	1 (0.7-1.4)	0.7 (0.3-1.2)
	2023	2.9 (2-4)	0.6 (0.4-1)	0.5 (0.2-1)
	2024	2.2 (0.9-3.5)	0.5 (0.3-1)	0.4 (0.2-0.9)
70% Concomitant HPV vaccination and screening	2020	4.1 (2.8-5.5)	2.1 (1-3)	0.9 (0.3-1.5)
	2021	3.5 (2.5-4.6)	1.9 (1-2.6)	0.8 (0.3-1.4)
	2022	3.2 (2.3-4.3)	0.8 (0.5-1.1)	0.6 (0.3-1.2)
	2023	2.9 (2-4)	0.4 (0.2-0.8)	0.5 (0.2-1)
	2024	2.2 (0.9-3.5)	0.4 (0.1-0.8)	0.4 (0.1-0.9)
90% Concomitant HPV vaccination and screening	2020	4.1 (2.8-5.5)	2.1 (1-3)	0.9 (0.3-1.5)
	2021	3.5 (2.5-4.6)	1.9 (1-2.6)	0.8 (0.3-1.4)
	2022	3.1 (2.3-4.2)	0.4 (0.2-0.7)	0.6 (0.2-1.2)
	2023	2.9 (1.9-3.9)	0.3 (0.1-0.5)	0.4 (0.1-1)
	2024	2.2 (0.9-3.4)	0.2 (0.1-0.5)	0.4 (0.1-0.8)

Note: 80% UI, 80% Uncertainty intervals.

STUDY
PROTOCOL

Study title:

Concomitant HPV vaccination and HPV screening for rapid elimination of HPV infection and cervical cancer in Sweden

EudraCT number: 2020-001169-34

Sponsor: **Region Stockholm**

Protocol version:
Version 2, date 2021-03-01

Participating sites iii
Signature Page iv
Amendments iv
Summary v
A. Rationale 7
A.1. HPV burden of disease and current cervical screening strategies 7
A.2 Integrating vaccination and screening – towards mid-adult vaccination programs 8
C. Methodology 10
C.1 Study design 10
C.2 Study target population 11
C.3 Study recruitment algorithms 11
C.4 Vaccine administration (Subsequent study visits) 12
C.5 Adverse events collection 12
C.6 Data collection and data management 12
A) Vaccine data recording 12
B) Data monitoring 13
C.7 Data analysis 13
C.8 Study limitations 14
D. Drug management 14
E. Ethical aspects and participating subjects protection 14
E.1 Ethical conduct of the trial 14
E.2 Benefit/risk assessement of participating subjects 15
E.3 Data confidentiality 15
E.4 Interference with physician's prescription 16
F. Adverse events management and communication 16
G. Results dissemination plan 21
H. Available resources to perform the study and assigned tasks. Funding. Drug supply. 21
H.1 Available resources and assigned tasks 21
H.2 Funding 22
H.3 Drug supply 22
I. CRITERIA FOR TERMINATION OF THE STUDY 22
J. Bibliography 22

PARTICIPATING SITES

The study will take place in several phases. The first phase merely evaluates the feasibility and logistics and will be performed in 11 Maternity Care units in the county of Stockholm, with the aim of enrolling approximately 7,000 women. The second phase will be a nationwide roll-out of the study over a phase of three years, aiming to enrol approximately 150,000 women. The third phase involves administration of a second dose of vaccine and the final phase is a long-term registry-based follow-up of effects.

Table 1. List of participating sites in phase 1. This list is complete for phase 1. These sites will also participate in phase 2 and 3. Selection of additional sites for phase 2 and 3 will be performed during phase 1.

Site	Centre
001	BBS Family Ekerö
002	Bromma BMM
003	Capio Wasa BMM
004	Farsta
005	Hammarby Sjöstads Barnmorskor
006	Märsta BMM
007	Sundbyberg
008	Tyresö BMM
009	Vantör
010	Vällingby BMM
011	Åkersberga BMM

SIGNATURE PAGE

Prof. Joakim Dillner
Sponsor
Karolinska University Hospital, F56
Clinical Patology and Cytology
141 86 Stockholm
+46 72 4682460
joakim.dillner@sll.se

Dr. Miriam Elfström
Investigator
Regional Cancer Center
Stockholm-Gotland
Box 6909, 102 39 Stockholm
+46 70 381 62 77
miriam.elfstrom@sll.se

AMENDMENTS

Amendment number	Date of amendment	Protocol version number	Type of amendment
1	2021-03-01	2	Specifications

Summary of Amendments

Clarification of endpoints, end of trial and SUSAR.

SUMMARY

STUDY IDENTIFICATION

Title:	**Concomitant HPV vaccination and HPV screening for rapid elimination of HPV infection and cervical cancer in Sweden**

RESPONSIBLE PERSONNEL AND PARTICIPATING SITES

This study will be conducted in all counties of Sweden, with the first feasibility phase being conducted in the county of Stockholm. The responsible study committee is the National Working Group for prevention of Cervical Cancer (NACx) (chaired by Miriam Elfström, PhD), appointed by the Regional Cancer Centers of Sweden. NACx has, in turn, appointed a task force for cervical cancer elimination, chaired by Joakim Dillner, who is also chairperson of the National Quality Registry for Cervical Cancer Prevention (NKCx) and ethically responsible Principle Investigator (PI). The sponsoring organisation is the county of Stockholm, Region Stockholm.

REGULATORY AND ETHICS APPROVALS

The PI will be responsible for identifying and obtaining the required approvals by the ethics committee and other authorities. Required approvals will be granted before study recruitment.

STUDY DESIGN AND METHODOLOGY

Up to 150,000 resident women aged 22-26 will be recruited for the main study. These women will be identified by the same method as is currently used by the organised cervical screening program of Sweden for identifying women who should receive an invitation for screening. The target group in this study is representative of all women in the population. The cumulative proportion of women aged 23-25 who participate in cervical screening in Sweden is >90%, an internationally uniquely high proportion. If such a high proportion of the population were to be HPV vaccinated, an extinction of oncogenic HPV infections in this population would follow. The first screening round in Sweden targets women aged 23-25 years of age, but we will accept women in the ages 22-26 - to avoid unnecessary exclusions.

Eligible women will receive an invitation to screening with concomitant vaccination. Women who do not consent to vaccination will receive the ordinary screening. Those who consent will receive the currently tendered HPV vaccine in Sweden Gardasil9® (MSD) and will be offered a second dose of the vaccine at their next visit for cervical screening. Efficiency and safety will be monitored by registry linkages. The study responsible committee (NACx) will receive such registry linkages for assessing safety of the trial on a yearly basis. For reporting of study endpoints, only endpoint assessments performed every 3 years will be used. Any medically responsible investigator at any of the enrolling sites can request a safety review at any time. The medically responsible clinician at each clinic must report serious adverse events to the sponsor who will report them to the Swedish Medical Products Agency (Läkemedelsverket, LVM). Additionally, all participating women will be informed that they can report any adverse events of vaccination that they experience to the sponsor and/or to LVM.

The study coordinating center will request a monthly aggregation of safety data reported to LVM that will be sent to all local sites, to increase awareness of any tendencies for adverse events seen. Annually, the study coordinating center will request from LVM individual-level data on adverse events reported by study participants.

CALENDAR

Enrolment in phase 1 (feasibility) is scheduled to start as early as possible in 2021 (depending on the COVID-19 pandemic situation) and in phase 2, enrolment will start as soon as possible, likely in 2022. The first formal reporting of study outcome is planned 3 years later, in 2025 and then subsequently, every 3 years until the end of trial.

FUNDING

The phase 1 of the study is funded by Region Stockholm. The funding of phase 2 is under discussion for public governmental funding. The infrastructure for the cervical screening program is funded by each participating county in Sweden. The infrastructure for follow-up (the national quality registry NKCx) is funded by the Swedish Association of Communities and Regions (SKR).

A. RATIONALE

A.1. HPV BURDEN OF DISEASE AND CURRENT CERVICAL SCREENING STRATEGIES

In Sweden, every year about 550 new cases of cervical cancer (CC) are detected and 150 women die from the disease. Globally, there are more than 500,000 new cases every year, implying that a success of the Swedish elimination study could have implications for the fight to elimination of cervical cancer also on a global scale. Other HPV-related cancers include those of the ano-genital tract and oropharynx, the incidences of which are increasing, and an elimination of HPV infection would thus also affect the incidences of these other HPV-associated cancers as well.

Countries with a well-organized screening program, and high screening participation, have observed a substantial decrease in CC incidence rates in the last decades whereas a disproportionally large number of cancer cases are observed in poorly screened and unscreened women. Recently, there has been an increase in CC cases in previously screened women in Sweden, indicating that a major nationwide campaign that optimally uses the available prevention tools will be required for an effective elimination.

Because of the risk for new oncogenic HPV infections and the suboptimal protection offered by the previously used screening tools, the organized screening program uses short screening intervals (3-7 years), leading to a substantial screening burden and costs. By a further elimination of HPV infection and a coordinated use of HPV screening we anticipate achieving an improved protection against CC, while at the same time enabling a prolongation of the screening intervals and a subsequent reduction of screening costs.

HPV DNA testing (the currently recommended cervical screening test) is more sensitive than the previously used cytology test to predict the presence of an underlying CIN2 lesion and shows less variability across populations (Marc Arbyn et al. 2006; Cuzick et al. 2006; Dillner et al. 2008). Evidence on the effectiveness of HPV screening comes from four large randomized screening trials performed in Sweden (Naucler et al. 2007), the UK (Kitchener et al. 2009), the Netherlands (Rijkaart et al. 2012), and Italy (Ronco et al. 2010) and the pooled analysis of the four RCT within Europe (Ronco et al. 2014). These studies have consistently shown that the sensitivity and predictive value for subsequent development of CIN2+ is higher with HPV testing than for cytology for intervals above 3-5 years. The high protection afforded by the HPV test is an important part of the elimination strategy that will be tried in the current study.

HPV vaccination is currently regarded as one of the most effective means of controlling HPV-related diseases. It has been shown to be over 95% efficacious in preventing CC, precancerous cervical lesions, and external genital lesions associated with HPV vaccine types in naïve populations (Schiller, Castellsagué, and Garland 2012).

In 2006, two vaccines were licensed to prophylactically protect against new HPV infections (Ault 2007; Lehtinen et al. 2012): a quadrivalent HPV6/11/16/18 vaccine Gardasil4 (Merck & Co, NJ USA) and a bivalent HPV16/18 vaccine Cervarix (GSK Biologicals, Rixensart, Belgium). Both

vaccines induce protection against the major cancer-causing HPV types, HPV16 and HPV18, responsible for approximately 70% of CC (Schiller, Castellsagué, and Garland 2012).

Currently, 93 countries have introduced HPV vaccination programs. Target ages for the vaccination are diverse, ranging from 9 to 26 years, although most programs prioritize vaccination of school-age girls.

However, at least 20 years would be required before cohorts vaccinated in schools will reach adult ages, with an expected effect on circulation of HPV, in turn reducing the burden of CC and other HPV-related diseases.

Studies have further indicated that catch-up vaccination up to the age of 26 may still be good value for money (Bogaards et al. 2011), which supports the use of catch-up vaccination programs for women beyond the age of 20. By May 2012, 10 out of 29 countries in the EU had introduced HPV vaccination catch-up programs (European Centre for Disease Prevention and Control 2012).

A.2 INTEGRATING VACCINATION AND SCREENING – TOWARDS MID-ADULT VACCINATION PROGRAMS

Recent results of Phase III vaccination trials have documented that vaccine efficacy among adult women (shown to age 45 for Gardasil and age 55 for Cervarix) is excellent (Table 2-3) (Castellsagué et al. 2011; Skinner et al. 2014). Therefore, the European Medicines Agency (EMA) has extended the indication of HPV vaccine to women aged 9+ with no upper age limit.

Table 2. Gardasil efficacy data (% reduction) against HPV 6/11/16/18-related outcomes in women aged 25-45 years, by analysis population.

	Per protocol	Naive to relevant type
**Any lesión**	88,7 (78,1-94,8)	79,9 (69,4-87,3)
**Persistent infection**	89,6 (79,3-95,4)	80,4 (69,9-87,7)
**CIN (any grade)**	94,1 (62,5-99,9)	89,0 (64,1-97,9)
**CIN2/3**	83,3 (-37,6-99,6)	62,7 (-55,5-93,6)
**External genital lesions**	100 (30,8-100)	81,9 (17,2-98,1)

Table 3. Cervarix preliminary efficacy data (% reduction) against HPV 16/18-related outcomes in women aged 26+ years, by analysis population.

	Per protocol	Naive to relevant type
**Any lesión**	81,1 (52,1-94,0)	74,0 (45,4-88,9)
**Persistent infection**	82,9 (53,8-95,1)	77,4 (49,7 to 91,1)
**CIN 1+**	86,1 (-35,4-99,9)	75,5 (–49,4 to 98,3)
CIN2/3	100 (-100,7-100,0)	80·4 (–125·3 to 99·8)

HPV vaccination coverage of adult women in Europe is currently low. Vaccination of women aged 22-26 could affect both

i) the acceleration of the reduction of CC mortality and
ii) the reduction of subsequent screening needs.

On 10th June 2015, of a broad-spectrum vaccine (Gardasil 9®; Sanofi Pasteur MSD SNC) that provides protection against 9 HPV types (7 oncogenic HPV types - 16, 18, 31, 33, 45, 52 and 58 - and HPV6/11,) responsible for 90% of cervical cancer cases was approved. The vaccine is indicated to individuals aged 9 or older based on high efficacy observed for the quadrivalent vaccine in women aged 16-45 and the comparable immunogenicity between Gardasil4 and Gardasil9 vaccines at ages 9-26 ('European Medicines Agency - Gardasil 9' 2015).The current study therefore wishes to study the population-level effect of free HPV vaccination invitation offered by the organised cervical cancer screening programs.

PRIMARY OBJECTIVE

The study aims to evaluate whether organised, concomitant HPV vaccination and HPV screening offered to all resident women aged 22-26 will result in a more rapid elimination of HPV infection in Sweden. This objective will be examined at the population level.

SECONDARY OBJECTIVES

The study will evaluate whether concomitant vaccination and cervical screening results in an improved efficiency and/or safety of the cervical screening program. These objectives will be examined among women who participated in combined screening and vaccination.

1) Protection of Gardasil 9 against HPV infection and against CIN2+ by Gardasil 9 HPV vaccine types. This analysis will be performed every third year by registry linkage with the NKCx. The first linkage will determine the effectiveness of one-dose vaccination, whereas all subsequent linkages will determine the effect of 2-dose vaccinations.
    a. Whether previous administration of first generation HPV vaccines (Gardasil 4) is an effect modifier will be examined.
2) Efficiency will be measured by the yield of histopathologically confirmed high-grade cervical cancer precursors or cancer (cervical intraepithelial neoplasia grade 2, 3, or cervical cancer) in relation to the consumption of resources and convenience for the women.
3) Safety will be measured by evaluating the occurrence of obstetrical complications such as preterm births as well as by measuring the number of excised cervical specimens found to be histopathologically benign.

Phase I of the protocol aims to evaluate and optimise logistics and feasibility. The personnel at all Phase I study sites will report on possible problems and suggest improvements. These will be evaluated by the study responsible committee (NACx) and if NACx decides that changes to the protocol are required, formal amendments will be made to the protocol.

ENDPOINTS

Endpoint	***Objective***	***Evaluation***
Primary: Prevalence of HPV infection in Sweden	To evaluate whether organised, concomitant HPV vaccination and HPV screening offered to all resident women aged 22-26	Overall and type-specific prevalence of HPV will be obtained from the routine HPV screening programs in

	will result in a more rapid elimination of HPV infection in Sweden	the counties that offer HPV-screening to this age group.
Secondary:<br>Histopathologically confirmed high-grade cervical cancer precursors or cancer (cervical intraepithelial neoplasia grade 2, 3, or cervical cancer) (CIN2+), by HPV type in the lesion.<br>Consumption of resources.<br>Number of screening and healthcare visits.<br>Obstetrical complications, in particular preterm births.<br>Excised cervical specimens found to be benign.	To evaluate whether the addition of concomitant vaccination to the cervical screening program results in an improved efficiency and/or safety of the cervical screening program	Registry linkages will determine the number of screening and treatment visits and their outcomes (CIN2+ lesions and benign excised cervical specimens) and obstetrical complications (e.g. preterm births).

END OF TRIAL

One screening interval (3 years) after the last visit of the last subject, defined as the day the last study subject receives her second vaccination, latest 2027-12-31.

C. METHODOLOGY

C.1 STUDY DESIGN

This is an open-label, multicentre and not controlled study to assess the efficiency and safety of concomitant HPV screening and HPV vaccination when offered by the organised screening program of Sweden.

In the feasibility phase, the study aims to optimise the delivery of the intervention.

The intervention is expected to be completed in 6 years in addition to the time required for the pilot study. There is a 3-year period to recruit the study participants and an additional 3 years to administrate a second screen and a second vaccine dose.

A table with main procedures is provided below:

	Visit 1	***Visit 2***	***Follow-up***
Timepoint	*M0*	*M36*	*Every 3 years*
Informed Consent	*X*		
HPV vaccine administration	*X*	*X*	
HPV vaccine recording	*X*	*X*	
Cervical screening (HPV testing)	*X*	*X*	*X*
Safety Data reporting (if applicable)	*X*	*X*	*X*

C.2 STUDY TARGET POPULATION

Eligible women will include resident women within the age range of 22-26, who have not opted out of the screening program and who consent to participate in the study.

Women who respond to the invitation and attend screening will be screened with HPV testing by the current routine practise. Women who consent to participate will also be offered HPV vaccination. The HPV vaccine (Gardasil 9) will be offered regardless of whether the woman reports having had prior vaccination with a first-generation vaccine (Gardasil 4) and regardless of screening test result.

Exclusion criteria for vaccination:

1. Known history of severe allergic reaction or hypersensitivity to any of the components of the HPV vaccine.
    - For *GARDASIL 9*: Amorphous aluminium hydroxyphosphate sulphate adjuvant, Sodium chloride, L-histidine, Polysorbate 80 or Sodium borate
2. Known history of immune-related disorders
3. Current acute severe febrile illness, except for minor infections such as a cold, mild upper respiratory infection or low-grade fever.
4. Administration of immunoglobulin or blood-derived products within 6 months prior to scheduled HPV vaccine first dose
5. Current pregnancy (reported)
6. Women with a total hysterectomy

Treatment withdrawal or temporary interruption

Women who develop symptoms indicative of hypersensitivity after receiving the initial HPV vaccine dose will not be administered the second HPV dose. The second dose will not be given in case of any serious adverse event.

In the event of acute illness (see exclusion criteria #3) or pregnancy, the subsequent dose will be delayed until resolution of illness or after pregnancy.

C.3 STUDY RECRUITMENT ALGORITHMS

Eligible resident women will be identified using the population registry. Women who have opted out of the screening program will be subtracted. The women will then be invited by letter, enclosing the patient information sheet containing information on the HPV vaccine and the study, and the study informed consent form. At the first visit, consenting women will hand over the signed informed consent form to the study personnel. Women who wish to participate but did not bring the consent form can fill it out on site, in that case the vaccine will be offered after signing the consent form for HPV vaccine administration.

The first dose will be administered at the same initial screening visit. If the woman requests time to think about HPV vaccine administration, to discuss it with whoever she considers necessary, or other action that would require an additional visit to get the first vaccine dose, this will be granted.

Informed consent will be obtained before vaccination. Before signing it, the patient information sheet will be provided to be read by the potential participant or explained by the study investigators and questions will be answered.

Workload: Independently of the procedure followed to recruit eligible women, each center will estimate how many women can be included per week based on their resources. This will allow monitoring of the study recruitment rate.

C.4 VACCINE ADMINISTRATION (SUBSEQUENT STUDY VISITS)

Gardasil 9® (Sanofi Pasteur MSD SNC):

HPV vaccine suspension of 0,5ml containing HPV type 6, 11, 16, 18, 31, 33, 45, 52 and 58 virus-like particles (VLPs), produced by recombinant DNA technology, and aluminium hydroxyphosphate sulphate as vaccine adjuvant. The vaccine will be ordered using routine clinical ordering practises.

It is to be administered as an intramuscular injection, preferably in the deltoid area of the upper arm or in the higher anterolateral area of the thigh.

Licensed indications, as approved by the EMA, include the use from the age of 9 years and upwards for the prevention of premalignant genital and cancers affecting the cervix, vulva, vagina and anus as well as genital warts

After each vaccine dose, women will be under observation for at least 15 minutes due to potential anaphylactic shock or fainting.

C.5 ADVERSE EVENTS COLLECTION

Any suspected unexpected serious adverse reactions as well as any potential immune mediated disease will be duly reported, if necessary, to health authorities as obliged by and following the adequate procedures detailed in section F.

C.6 DATA COLLECTION AND DATA MANAGEMENT

A) Vaccine data recording

For those women who accept the vaccine, date of HPV vaccine administration, dose, and batch number will be recorded in the woman's medical chart. The lab processing the cervical sample will be notified that the women has been included in the study and been vaccinated with Gardasil 9 on the referral form for cervical screening.

B) Data monitoring

Periodic checks will be performed on collected data and issue-related queries would be resolved by investigators. Data can be corrected if necessary, but any modification will be tracked.

The monitor, certain regulatory personnel and representatives from authorities must have direct access to the source data/records for monitoring and inspections, and this will be granted so by the investigators (and patients).
The monitoring will be performed by an independent experienced monitor qualified in ICH GCP, applicable national and international regulations, and the Declaration of Helsinki.

The database containing all the anonymised study information will be stored according to current data protection regulation, as detailed in section F3.

By means of the study web-based platform, centers will be periodically informed on national study progression regarding sites recruitment, vaccine uptake, and study completion.

C.7 DATA ANALYSIS

PRIMARY OBJECTIVE - ELIMINATION OF HPV INFECTION

To evaluate whether concomitant HPV vaccination and HPV screening results in more rapid elimination of HPV infection in Sweden, a time trend analysis will be used. This population-based, ecological design allows for rapid evaluation of the intervention based on routine screening data.

HPV prevalence overall and type-specific (7 oncogenic types included Gardasil 9) will be evaluated among 26-year-old women using screening test results. A joinpoint analysis of trends will be used assess non-linearity in prevalence over time (yearly measurements). This analysis method is appropriate given that it can accommodate abrupt changes in trends as would be expected following the introduction of Gardasil 9 vaccination for women entering screening.

SECONDARY OBJECTIVES – EFFECTIVENESS, EFFICIENCY, AND SAFETY

To evaluate effectiveness, efficiency, and safety, women who participated in vaccination and screening at ages 23-25 will be examined in an individual level cohort analysis. Linkages to vaccine registers to obtain prior vaccination status with Gardasil 4 will be completed and evaluated as a potential effect modifier in the analyses described below. A comparison group of women who entered the screening program prior to this intervention will be used to determine the relative contribution of concomitant vaccination and screening. Registry linkages will be used to examine screening and treatment outcomes (CIN2+ lesions and benign excised cervical specimens extracted from NKCx, Cytburken, and Quality Register for Gynecological Cancers) and obstetrical complications (e.g. preterm births, extracted from the Medical Birth Registry). The first analysis will examine 1 dose effectiveness. Subsequent analyses will include both doses.

LOGISTICS AND FEASIBILITY

Phase I of the protocol, the implementation of the study in the region of Stockholm with a limited number of participants, will be focused on evaluating logistics and feasibility of the concept. Interviews and -Phase I of the protocol aims to evaluate and optimise logistics and feasibility. The personnel at all Phase I study sites will report on possible problems and suggest improvements. These will be evaluated by the study responsible committee (NACx) and if NACx decides that changes to the protocol are required, formal amendments will be made to the protocol.

C.8 STUDY LIMITATIONS

The evaluation of the first study objective will be done at the population level. While these methods do not involve individual level linkages and therefore cannot determine causality, they are appropriate given the timeframe of the study and the need for timely analyses of the impact of vaccination in conjunction with screening.

The analyses planned for the secondary study objectives will involve comparisons with historical cohorts in a before/after design; such designs have limitations. However, this analysis strategy has been deemed to be the most applicable since withholding vaccination and creating a control group would be unethical given our knowledge of vaccine performance.

D. DRUG MANAGEMENT

HPV vaccines will be directly delivered from manufacturing companies to study sites for drug storage, labelling and accountability.

HPV vaccine storage must follow the internal regulation and logistics. Vaccine must be kept under temperature regulations thus each participant center needs to check the flow and storage conditions at the study office/center.

According to the EU clinical trial directive 2001/20/EC, special provisions might be adopted by each EU country for trials using medicinal products under a marketing authorization. Adequate documentation, traceability, accountability and labelling will be followed as per country requirements and study classification.

E. ETHICAL ASPECTS AND PARTICIPATING SUBJECTS PROTECTION

E.1 ETHICAL CONDUCT OF THE TRIAL

The study will be conducted according to the protocol and in line with applicable national and international regulations, ICH GCP and the latest version of the Declaration of Helsinki from 2013.

Deviations from the protocol are to be made in the form of substantial amendments, which must be approved by the Competent Authorities, Ethics Committees /IRB's and Sponsor. Amendments that are considered as substantial are changes that are likely to have an impact on

the safety of the trial subject or to change the interpretation of the scientific documents in support of the conduct of the trial, or if they are otherwise significant.

The Principal Investigator and all investigators involved in the trial will have to sign an Investigator protocol signature page to verify that they will conduct the study in accordance with the latest version of the study protocol, ICH GCP, applicable national and international regulations and the Declaration of Helsinki. Any changes in the protocol will not be valid until approved by the sponsor, the Competent Authorities and Ethics Committees/IRB's.

E.2 BENEFIT/RISK ASSESSEMENT OF PARTICIPATING SUBJECTS

Receiving HPV vaccination will add to the screening effect the following protection, depending on HPV type presence. Women HPV negative to all carcinogenic HPV vaccine types will gain an estimated >90% protection against HPV vaccine types infection, persistent infection and CIN 2. Women positive to any of the HPV vaccine types (expected prevalence HPV16: 2-3% and remaining HPV vaccine types: <1%, will gain an estimated (estimated >85%) protection against the other HPV type.

Data from clinical trials has shown that vaccination of HPV positive women does not alter the HPV infection prognosis and does not convey safety concerns.

Concerning risk assessment connected to the COVID-19 outbreak, the study population does not belong to a risk-group as far as age is concerned. Some COVID-19 risk categories, for instance immuno-compromised individuals, are excluded from the study. The study subjects will only be called to participate with an invitation to a given timeslot to avoid crowding when screened and vaccinated and the flow of participants entering and leaving the study sites is designed to avoid unnecessary encounters (same design as is currently used for Covid 19 vaccinations will be used) Healthcare workers performing the screening and vaccinations will as far as possible be vaccinated and are equipped with COVID-19 protection outfit.

No risks are expected other than those related to any vaccine administration and those detailed in the summary of product characteristics.

E.3 DATA CONFIDENTIALITY

Analysis, electronic management and reporting or dissemination of data and results of this study will be done in compliance to national and EU legislation. Specific EU legislation includes the European Directive on Data Protection 95/46/EC in relation to medical research (privacy protection), the European Directive 95/46/EC (amendment 2003) on protection of privacy, and the Regulation (CE) No 45/2001 of the European Parliament and of the Council of 18 December 2001, on privacy protection of individuals.

In case of discrepancy between national and EU legislation, whichever is more restrictive will be followed.

The ethical and data protection objectives of the study will ensure that:

1. The data anonymization, pseudonymization or de-identification processes in place are fit for purpose and conform to the study informed consent;
2. There are no issues with unauthorized cross border access to the data;
3. All investigators shall have access to the data that they have generated;
4. Access to data by users other than the data owners shall be determined by the conditions imposed by the informed consent of the patient;
5. Access to data may also be restricted at the request of the owner to specified groups of users, for example, in order to protect intellectual property rights;
6. Under no circumstances shall unauthorized persons have access to the data.

The risk of patient-level data being released accidentally will be minimized by implementing risk control assessment at the source. Any transmission of information via electronic means (e.g. between data centres) will only be performed using advanced encryption.

E.4 INTERFERENCE WITH PHYSICIAN'S PRESCRIPTION

The HPV vaccines are prophylactic drugs currently marketed and available to all individuals older than 9 years old.

Because of its prevention indication and lack of public funding to administer it to adult women, it is not actually being prescribed. However, the vaccine will only be offered to those women whom the investigators might consider that could get a benefit, as previously established regarding age range and screen results restrictions (Table 4).

F. ADVERSE EVENTS MANAGEMENT AND COMMUNICATION

Serious Adverse Events should be reported from the Investigator to the sponsor within 24 hours.

Among all the collected adverse events, active search for any suspected unexpected serious adverse reactions (SUSAR) and potential immune mediated diseases (pIMD) will be performed.

A SUSAR is defined as a serious adverse drug reaction for which have a reasonable possibility of a causal relationship to the investigational product are considered unexpected. Therefore, all causally related serious events will be reported as SUSAR's to Competent Authorities, Ethics Committees / IRB's and investigators, as required.

	**GARDASIL**
**System Organ Class**	**Adverse Events**
Infections and infestations	*Unknown:* Injection-site cellulitis *
Blood and lymphatic system disorders	*Unknown:* Idiopathic thrombocytopenic purpura*, lymphadenopathy*

Immune system disorders	*Unknown:* Hypersensitivity reactions including anaphylactic/anaphylactoid reactions*
Nervous system disorders	*Very common:* Headache<br>*Common:* Dizziness<br>*Unknown:* Guillain-Barré syndrome*,<br>syncope sometimes accompanied by tonic-clonic movements*
Gastrointestinal disorders	*Common:* Nausea<br>*Unknown:* Vomiting*
Musculoskeletal and Connective Tissue Disorders	*Common:* Pain in extremity<br>*Unknown:* Arthralgia*, Myalgia*
General disorders and administration site conditions	*Very common:* At the injection site: erythema, pain, swelling<br>*Common:* Pyrexia and at the injection site: hematoma, pruritus<br>*Unknown:* Asthenia*, chills*, fatigue*, malaise*
Skin and subcutaneous tissue disorders	

* Post Marketing adverse events (frequency cannot be estimated from the available data).
Frequencies are reported as: Very common (≥1/10); Common (≥1/100 to <1/10); Uncommon (≥1/1,000 to <1/100)

	**GARDASIL 9**
**System Organ Class**	**Adverse Events**
Nervous system disorders	*Very common:* Headache<br>*Common:* Dizziness
Gastrointestinal disorders	*Common:* Nausea
General disorders and administration site conditions	*Very common:* At the injection site: erythema, pain, swelling<br>*Common:* Pyrexia, fatigue and at the injection site: pruritus and bruising
Because of the similar composition of both vaccines, those adverse events observed or reported during the post-marketing evaluation of Gardasil might occur in women vaccinated with Gardasil 9.	

A pIMD is defined as a subset of immune mediated inflammatory disorders which may or may not have an autoimmune etiology. The mechanisms underlying immune mediated disorders are diverse, complex and not fully understood. Therefore, in Appendix 8, a list of pIMD is provided as a reference, including any disease for which an autoimmune-dependent mechanism has been postulated even if not established (Tavares Da Silva et al. 2013).

Immediate Reporting of Adverse Experiences to the sponsor

Serious Adverse Experiences

Any serious adverse experience, including death, related to protocol-specified procedures, which occurs to any patient from the time the consent is signed through 24 hours following the

first visit and from the time of any subsequent visit through 24 hours thereafter, must be reported within 24 hours of investigator becoming aware to the sponsor.

Additionally, any serious adverse experience related to protocol-specified procedures that is brought to the attention of the investigator at any time outside of the time period specified in the previous paragraph or a serious adverse experience considered by an investigator who is a qualified physician to be possibly, probably, or definitely related to the vaccination must be reported immediately to the sponsor.

All patients with serious adverse experiences related to protocol-specified procedures or possibly, probably or definitely related to vaccination must be followed-up for outcome. Follow-up must continue until the serious adverse event is resolved or stabilized.

Other adverse experiences will not be ascertained or evaluated in this protocol.

Evaluating Adverse Experiences

Serious adverse experiences related to protocol-specified procedures will be collected as described in the previous paragraph. Within the context of this study, an adverse experience (AE) is defined as any unfavorable and unintended change in the structure, function, or chemistry of the body temporally associated with the protocol-specified procedure whether or not considered related to the procedure. Any worsening (i. e. any clinically significant adverse change in frequency and/or intensity) of a preexisting condition which is temporally associated with the protocol-specified procedure, is also an adverse experience.

An investigator, who is a qualified physician, will evaluate all adverse experiences as to:

- Mild (awareness of sign or symptom, but easily tolerated);
- Moderate (discomfort enough to cause interference with usual activity);
- Severe (incapacitating with inability to work or do usual activity)

- Seriousness:

A serious adverse experience is any adverse experience that:

- †Results in death; or
- †Is life threatening (places the subject/patient, in the view of the investigator, at immediate risk of death from the experience as it occurred [Note: This does not include an adverse experience that, had it occurred in a more severe form, might have caused death.]); or
- †Results in a persistent or significant disability/incapacity (substantial disruption of one´s ability to conduct normal life functions); or
- †Results in or prolongs an existing inpatient hospitalization (hospitalization is defined as an inpatient admission, regardless of length of stay, even if the hospitalization is a precautionary measure for continued observation) (Note: Hospitalization [including hospitalization for an elective procedure] for a preexisting condition which has not worsened does not constitute a serious adverse experience); or

- †Is a congenital anomaly/birth defect (in offspring of subject/patient taking the product regardless of time to diagnosis); or
- Is a cancer; or
- Is an overdose (whether accidental or intentional)

N. B. Any overdose whether or not associated with an adverse experience must be reported within 24 hours to the sponsor.

ALSO:

Other important medical events that may not result in death, not be life threatening, or not require hospitalization may be considered a serious adverse experience when, based upon appropriate medical judgement, the event may jeopardize the subject/patient and may require medical or surgical intervention to prevent one of the outcomes listed previously (designated above by a †).

- Duration:

Record the start and stop dates of the adverse experience. If less than 1 day, indicate the appropriate length of time and units.

- Action taken (Did the adverse experience cause the protocol-specified procedure to be discontinued?); and
- Relationship to protocol-specified procedures (Did the protocol-specified procedure cause the adverse experience?):

The determination of the likelihood that the protocol-specified procedure caused the adverse experience will be provided by an investigator who is a qualified physician. The investigator´s signed/dated initials on the source document or worksheet, that supports the causality noted on the AE form, ensure that a medically qualified assessment of causality was done. This initialed document must be retained for the required regulatory time frame. The criteria below are intended as reference guidelines to assist the investigator in assessing the likelihood of a relationship between the protocol-specified procedure and the adverse experience based upon the available information.

The following components are to be used to assess this relationship; the greater the correlation with the components and their respective elements (in number and/or intensity), the more likely the protocol-specified procedure caused the adverse experience:

- Exposure:

Is there evidence that the subject/patient actually underwent the protocol-specified procedure?

- Time Course:

Did the AE follow in a reasonable temporal sequence form conduct of the protocol-specified procedure?
Is the time of onset of the AE compatible with an effect of the protocol-specified procedure?

- Likely Cause:

Is the AE not reasonably explained by another etiology such as underlying disease, other drug(s), or other host or environmental factors?

- Consistency With Profile of the protocol-specified procedure:

Is the clinical/pathological presentation of the AE consistent with previous knowledge regarding the protocol-specified procedure?

The assessment of relationship will be reported on the case report forms/worksheets by an investigator who is a qualified physician according to his/her best clinical judgement, including consideration of the above elements. Use the following scale of criteria as guidance (not all criteria must be present to be indicative of a relationship to a protocol-specified procedure).

- Definitely related to the protocol-specified procedure:

There is evidence of exposure to the protocol-specified procedure.
The temporal sequence of the AE onset relative to conduct of the protocol-specified procedure is reasonable.
The AE is more likely explained by the protocol-specified procedure than by another cause.
The AE shows a pattern consistent with previous knowledge of the protocol-specified procedure.

- Probably related to protocol-specified procedure:

There is evidence of exposure to the protocol-specified procedure.
The temporal sequence of the AE onset relative to conduct of the protocol-specified procedure is reasonable.
The AE is more likely explained by the protocol-specified procedure than by another cause.

- Possibly related to protocol-specified procedure:

There is evidence of exposure to the protocol-specified procedure.
The temporal sequence of the AE onset relative to administration of the protocol-specified procedure is reasonable.
The AE could have been due to another equally likely cause.

- Probably not related to protocol-specified procedure:

There is evidence of exposure to the protocol-specified procedure.
There is another more likely cause of the AE.

- Definitely not related to protocol-specified procedure:

The subject/patient did not undergo the protocol-specified procedure.

OR

Temporal sequence of the AE onset relative to conduct of the protocol-specified procedure is not reasonable.

OR

There is another obvious cause of the AE.

Serious adverse experiences will be reported to regulatory agencies, IRB/IECs, and investigators in accordance with all applicable global laws and regulations.

In case of suspicion of SUSAR or pIMD, the sponsor will be notified as soon as possible after awareness by the participating sites. The sponsor will inform the respective manufacturer in his country to ensure Regulatory Authorities reporting. If necessary, additional information will be collected for an adequate reporting

Upon manufacturer confirmation, safety reporting will be performed by study participants according to national established procedures for reporting of post-marketing adverse events. The sponsor will be provided with a copy of the report sent, who will forward it to the manufacturer safety department.

G. RESULTS DISSEMINATION PLAN

Irrespective of the results obtained in the primary and secondary endpoints, these will be disseminated by publishing in international and peer-reviewed journals, using other channels of publications (e.g. through reports or white papers) and/or presented at relevant events, congresses, courses, and meetings.

We will strive to ensure free access to peer-reviewed articles resulting from this study, which will optimize knowledge transfer.

H. AVAILABLE RESOURCES TO PERFORM THE STUDY AND ASSIGNED TASKS. FUNDING. DRUG SUPPLY.

H.1 AVAILABLE RESOURCES AND ASSIGNED TASKS

Personnel, equipment and facilities used in the study will be provided by participating sites. Furthermore, participating sites will be responsible for recruitment of women, vaccine stock and administration, and data entry into medical charts and clinical referrals. Field work burden per woman for study sites personnel has been estimated as 15' to explain the study (in clinics), 20' for initial vaccine dose, 10' for the second dose, and 5' to report vaccination.

Region Stockholm will be responsible for database creation and management, data monitoring and final data analyses. The responsible scientists will write the final report with the main study results.

H.2 FUNDING

The phase 1 of the study is funded by Region Stockholm through the Regional Cancer Centre Stockholm Gotland. The funding of phase 2 is under discussion for public governmental funding. The infrastructure for enrolment (cervical screening program) is funded by each participating county in Sweden. The infrastructure for follow-up (the national quality registry NKCx) is funded by the Swedish Association of Communities and Regions (SKR).

H.3 DRUG SUPPLY

HPV vaccines will be purchased from Sanofi Pasteur MSD SNC through a direct tendering process and distributed to participating sites.

I. CRITERIA FOR TERMINATION OF THE STUDY

The sponsor reserves the right to terminate the study prematurely due to scientific, administrative and/or ethical reasons. The study may be terminated prematurely if unexpectedly high events of reported unexpected serious adverse events or if the recruitment of subjects is out of reasonable timelines. The decision of early termination is done by sponsor.

J. BIBLIOGRAPHY

Arbyn, Marc, Jerome Antoine, Margit Mägi, Giedre Smailyte, Aivars Stengrevics, Ofelia Suteu, Zdravka Valerianova, Freddie Bray, and Elisabete Weiderpass. 2011. 'Trends in Cervical Cancer Incidence and Mortality in the Baltic Countries, Bulgaria and Romania'. *International Journal of Cancer* 128 (8): 1899–1907. doi:10.1002/ijc.25525.

Arbyn, Marc, Peter Sasieni, Chris J L M Meijer, Christine Clavel, George Koliopoulos, and Joakim Dillner. 2006. 'Chapter 9: Clinical Applications of HPV Testing: A Summary of Meta-Analyses'. *Vaccine* 24 Suppl 3 (August): S3/78–89. doi:10.1016/j.vaccine.2006.05.117.

Arbyn, M, A O Raifu, P Autier, and J Ferlay. 2007. 'Burden of Cervical Cancer in Europe: Estimates for 2004'. *Annals of Oncology: Official Journal of the European Society for Medical Oncology / ESMO* 18 (10): 1708–15. doi:10.1093/annonc/mdm079.

Ault, Kevin A. 2007. 'Effect of Prophylactic Human Papillomavirus L1 Virus-like-Particle Vaccine on Risk of Cervical Intraepithelial Neoplasia Grade 2, Grade 3, and Adenocarcinoma in Situ: A Combined Analysis of Four Randomised Clinical Trials'. *Lancet* 369 (9576): 1861–68. doi:10.1016/S0140-6736(07)60852-6.

Bogaards, Johannes A, Veerle M H Coupé, Chris J L M Meijer, and Johannes Berkhof. 2011. 'The Clinical Benefit and Cost-Effectiveness of Human Papillomavirus Vaccination for Adult Women in the Netherlands'. *Vaccine* 29 (48): 8929–36. doi:10.1016/j.vaccine.2011.09.055.

Castellsagué, X, N Muñoz, P Pitisuttithum, D Ferris, J Monsonego, K Ault, J Luna, et al. 2011. 'End-of-Study Safety, Immunogenicity, and Efficacy of Quadrivalent HPV (types 6, 11, 16, 18) Recombinant Vaccine in Adult Women 24-45 Years of Age'. *British Journal of Cancer*, May. doi:10.1038/bjc.2011.185.

Cuzick, Jack, Christine Clavel, Karl-Ulrich Petry, Chris J L M Meijer, Heike Hoyer, Samuel Ratnam, Anne Szarewski, et al. 2006. 'Overview of the European and North American Studies on HPV Testing in Primary Cervical Cancer Screening'. *International Journal of Cancer. Journal International Du Cancer* 119 (5): 1095–1101. doi:10.1002/ijc.21955.

Dillner, Joakim, Matejka Rebolj, Philippe Birembaut, Karl-Ulrich Petry, Anne Szarewski, Christian Munk, Silvia de Sanjose, et al. 2008. 'Long Term Predictive Values of Cytology and

Human Papillomavirus Testing in Cervical Cancer Screening: Joint European Cohort Study'. *BMJ (Clinical Research Ed.)* 337: a1754.

European Centre for Disease Prevention and Control. 2012. 'Introduction of HPV Vaccines in EU Countries – an Update'. Stockholm: ECDC.

'European Medicines Agency - Gardasil 9'. 2015. Accessed July 13. http://www.ema.europa.eu/ema/index.jsp?curl=pages/medicines/human/medicines/003852/human_med_001863.jsp&mid=WC0b01ac058001d124.

Kitchener, Henry C, Maribel Almonte, Claire Thomson, Paula Wheeler, Alexandra Sargent, Boyka Stoykova, Clare Gilham, et al. 2009. 'HPV Testing in Combination with Liquid-Based Cytology in Primary Cervical Screening (ARTISTIC): A Randomised Controlled Trial'. *The Lancet Oncology* 10 (7): 672–82. doi:10.1016/S1470-2045(09)70156-1.

Lehtinen, Matti, Jorma Paavonen, Cosette M Wheeler, Unnop Jaisamrarn, Suzanne M Garland, Xavier Castellsagué, S Rachel Skinner, et al. 2012. 'Overall Efficacy of HPV-16/18 AS04-Adjuvanted Vaccine against Grade 3 or Greater Cervical Intraepithelial Neoplasia: 4-Year End-of-Study Analysis of the Randomised, Double-Blind PATRICIA Trial'. *The Lancet Oncology* 13 (1): 89–99. doi:10.1016/S1470-2045(11)70286-8.

Naucler, Pontus, Walter Ryd, Sven Törnberg, Anders Strand, Göran Wadell, Kristina Elfgren, Thomas Rådberg, et al. 2007. 'Human Papillomavirus and Papanicolaou Tests to Screen for Cervical Cancer'. *The New England Journal of Medicine* 357 (16): 1589–97. doi:10.1056/NEJMoa073204.

Rijkaart, Dorien C, Johannes Berkhof, Lawrence Rozendaal, Folkert J van Kemenade, Nicole WJ Bulkmans, Daniëlle AM Heideman, Gemma G Kenter, Jack Cuzick, Peter JF Snijders, and Chris JLM Meijer. 2012. 'Human Papillomavirus Testing for the Detection of High-Grade Cervical Intraepithelial Neoplasia and Cancer: Final Results of the POBASCAM Randomised Controlled Trial'. *The Lancet Oncology* 13 (1): 78–88. doi:10.1016/S1470-2045(11)70296-0.

Ronco, Guglielmo, Joakim Dillner, K Miriam Elfström, Sara Tunesi, Peter J F Snijders, Marc Arbyn, Henry Kitchener, et al., International HPV screening working group. 2014. 'Efficacy of HPV-Based Screening for Prevention of Invasive Cervical Cancer: Follow-up of Four European Randomised Controlled Trials'. *Lancet* 383 (9916): 524–32. doi:10.1016/S0140-6736(13)62218-7.

Ronco, Guglielmo, Paolo Giorgi-Rossi, Francesca Carozzi, Massimo Confortini, Paolo Dalla Palma, Annarosa Del Mistro, Bruno Ghiringhello, et al. 2010. 'Efficacy of Human Papillomavirus Testing for the Detection of Invasive Cervical Cancers and Cervical Intraepithelial Neoplasia: A Randomised Controlled Trial'. *The Lancet Oncology* 11 (3): 249–57. doi:10.1016/S1470-2045(09)70360-2.

Schiller, John T, Xavier Castellsagué, and Suzanne M Garland. 2012. 'A Review of Clinical Trials of Human Papillomavirus Prophylactic Vaccines'. *Vaccine* 30 Suppl 5 (November): F123–38. doi:10.1016/j.vaccine.2012.04.108.

Skinner, S. Rachel, Anne Szarewski, Barbara Romanowski, Suzanne M. Garland, Eduardo Lazcano-Ponce, Jorge Salmerón, M. Rowena Del Rosario-Raymundo, et al., VIVIANE Study Group. 2014. 'Efficacy, Safety, and Immunogenicity of the Human Papillomavirus 16/18 AS04-Adjuvanted Vaccine in Women Older than 25 Years: 4-Year Interim Follow-up of the Phase 3, Double-Blind, Randomised Controlled VIVIANE Study'. *Lancet* 384 (9961): 2213–27. doi:10.1016/S0140-6736(14)60920-X.

Tavares Da Silva, Fernanda, Filip De Keyser, Paul-Henri Lambert, William H. Robinson, René Westhovens, and Christian Sindic. 2013. 'Optimal Approaches to Data Collection and Analysis of Potential Immune Mediated Disorders in Clinical Trials of New Vaccines'. *Vaccine* 31 (14): 1870–76. doi:10.1016/j.vaccine.2013.01.042.

Wheeler, Cosette M, Xavier Castellsagué, Suzanne M Garland, Anne Szarewski, Jorma Paavonen, Paulo Naud, Jorge Salmerón, et al. 2012. 'Cross-Protective Efficacy of HPV-16/18 AS04-Adjuvanted Vaccine against Cervical Infection and Precancer Caused by Non-Vaccine

Oncogenic HPV Types: 4-Year End-of-Study Analysis of the Randomised, Double-Blind PATRICIA Trial.' *The Lancet Oncology* 13 (1): 100–110. doi:10.1016/S1470-2045(11)70287-X.

Signature page

This document has been electronically signed using eduSign.

eduSign

Validate signed documents at: https://validator.edusign.sunet.se

SUNET
