# Supplementary material for: Concomitant human papillomavirus (HPV) vaccination and screening for elimination of HPV and cervical cancer
Source: Nat Commun. 2024 May 1;15:3679. doi: 10.1038/s41467-024-47909-x (PMC11063066; doi:10.1038/s41467-024-47909-x)
Supplement: Supplementary file 3 — Reporting Summary [file 41467_2024_47909_MOESM3_ESM.pdf]

nature portfolio

Corresponding author(s): Joakim Dillner

Last updated by author(s): Apr 9, 2024

Reporting Summary

Nature Portfolio wishes to improve the reproducibility of the work that we publish. This form provides structure for consistency and transparency in reporting. For further information on Nature Portfolio policies, see our Editorial Policies and the Editorial Policy Checklist.

Statistics

For all statistical analyses, confirm that the following items are present in the figure legend, table legend, main text, or Methods section.

n/a	Confirmed	
☐	☒	The exact sample size (n) for each experimental group/condition, given as a discrete number and unit of measurement
☐	☒	A statement on whether measurements were taken from distinct samples or whether the same sample was measured repeatedly
☒	☐	The statistical test(s) used AND whether they are one- or two-sided<br>*Only common tests should be described solely by name; describe more complex techniques in the Methods section.*
☒	☐	A description of all covariates tested
☒	☐	A description of any assumptions or corrections, such as tests of normality and adjustment for multiple comparisons
☒	☐	A full description of the statistical parameters including central tendency (e.g. means) or other basic estimates (e.g. regression coefficient) AND variation (e.g. standard deviation) or associated estimates of uncertainty (e.g. confidence intervals)
☐	☒	For null hypothesis testing, the test statistic (e.g. F, t, r) with confidence intervals, effect sizes, degrees of freedom and P value noted<br>*Give P values as exact values whenever suitable.*
☒	☐	For Bayesian analysis, information on the choice of priors and Markov chain Monte Carlo settings
☒	☐	For hierarchical and complex designs, identification of the appropriate level for tests and full reporting of outcomes
☒	☐	Estimates of effect sizes (e.g. Cohen's d, Pearson's r), indicating how they were calculated

Our web collection on statistics for biologists contains articles on many of the points above.

Software and code

Policy information about availability of computer code

Data collection	The input data for the modelling was obtain from the website Swedish government authorities: Data available at https://www.folkhalsomyndigheten.se/folkhalsorapportering-statistik/statistikdatabaser-och-visualisering/vaccinationsstatistik/statistik-for-hpv-vaccinationer/ and https://www.folkhalsomyndigheten.se/barnvaccinationer/, accessed on 25th August 2023).
Data analysis	The code used in modelling analysis is openly available and can be found at https://gitlab.com/iarc-miarc/analysis/concomitant-hpv.

For manuscripts utilizing custom algorithms or software that are central to the research but not yet described in published literature, software must be made available to editors and reviewers. We strongly encourage code deposition in a community repository (e.g. GitHub). See the Nature Portfolio guidelines for submitting code & software for further information.

Data

Policy information about availability of data

All manuscripts must include a data availability statement. This statement should provide the following information, where applicable:

- Accession codes, unique identifiers, or web links for publicly available datasets
- A description of any restrictions on data availability
- For clinical datasets or third party data, please ensure that the statement adheres to our policy

The data generated in this study have been deposited in the Swedish National Cervical Screening Registry database under accession code 2020-001169-34 [www.nkcx.se]. The data are available under restricted access for research use. Access can be obtained by applying to the registry as described on www.nkcx.se (an

Institutional Review Board approval must be obtained and a Data Use Agreement must be completed, no other restrictions apply). The raw indivdidual data are protected and are not available due to data privacy laws. The processed data are available at www.nkcx.se. The data generated in this study are provided in the Supplementary Information.

Research involving human participants, their data, or biological material

Policy information about studies with human participants or human data. See also policy information about sex, gender (identity/presentation), and sexual orientation and race, ethnicity and racism.

Reporting on sex and gender	Cervical cancer is restricted to subjects of the female sex and transgender males. The population registry specifies the sex of the individual and the sex registered in the population registry is included in the personal identification number that is stated on all issued ID documents in Sweden and was collected from all enrolled subjects. All subjects with a registered female sex born 1999-1994 who provided informed consent were eligible for the study. Transgender males (subjects born as females, but having changed sex) were not eligible as the protocol had specified females. However, transgender males received the vaccine and the screening as a compassionate out-of-study measure. As all data refers to subjects of the female sex, sex-stratified data is not presented.
Reporting on race, ethnicity, or other socially relevant groupings	NA
Population characteristics	All subjects in the entire population were eligible if they were of female sex, born 1999-1994 and provided informed consent, did not have contraindications to vaccination and were not hysterectomised. Invitations used the actual population registry and the population characteristics of the study is thus identical to the population characteristics of Sweden.
Recruitment	Population-based invitations were issued to the entire population, both by electronic push messages, SMS and physical letters. We used 2 concomitant strategies: 1. The women could book a convenient time and place themselves ("campaign") or 2. A time and place was included in the invitation ("organised screening"). This is described in the manuscript in detail in the methods. Possible biases by determinants of attendance can not be excluded. However, as a large part of the population was enrolled it is likely that the enrolled subjects are representative of the population.
Ethics oversight	The Swedish National Ethical Review Agency (a government agency) and the Swedish Medical Products Agency.

Note that full information on the approval of the study protocol must also be provided in the manuscript.

Field-specific reporting

Please select the one below that is the best fit for your research. If you are not sure, read the appropriate sections before making your selection.

☒ Life sciences ☐ Behavioural & social sciences ☐ Ecological, evolutionary & environmental sciences

For a reference copy of the document with all sections, see nature.com/documents/nr-reporting-summary-flat.pdf

Life sciences study design

All studies must disclose on these points even when the disclosure is negative.

Sample size	As the aim was faster cervical cancer elimination in the population of Sweden, the entire population was targeted
Data exclusions	Lack of informed consent and contraindication to HPV vaccination (allergy to components of the vaccine, pregnancy).
Replication	The HPV testing was performed by the Swedish cervical screening program used the cervical screening program quality assurance system. This involves a series of quality checks for proficiency including reproducibility. Separate repeat analysis for the samples in this study was not done. The overall proficiency of the testing is published.
Randomization	The study targeted all women. A control group was not ethical, as vaccination and screening are known to prevent cancer. Evaluation is therefore using a before-after design.
Blinding	The study targeted all women. A control group was not ethical, as vaccination and screening are known to prevent cancer. Evaluation is therefore using a before-after design.

Reporting for specific materials, systems and methods

We require information from authors about some types of materials, experimental systems and methods used in many studies. Here, indicate whether each material, system or method listed is relevant to your study. If you are not sure if a list item applies to your research, read the appropriate section before selecting a response.

Materials & experimental systems

n/a	Involved in the study
☒	☐ Antibodies
☒	☐ Eukaryotic cell lines
☒	☐ Palaeontology and archaeology
☒	☐ Animals and other organisms
☐	☒ Clinical data
☒	☐ Dual use research of concern
☒	☐ Plants

Methods

n/a	Involved in the study
☒	☐ ChIP-seq
☒	☐ Flow cytometry
☒	☐ MRI-based neuroimaging

Clinical data

Policy information about clinical studies

All manuscripts should comply with the ICMJE guidelines for publication of clinical research and a completed CONSORT checklist must be included with all submissions.

Clinical trial registration	Clinicaltrials.gov identifier: NCT04910802; EudraCT number: 2020-001169-34
Study protocol	The full protocol is available in the open repositories of the Swedish National Ethical Review Agency and the Swedish Medical Products Agency.
Data collection	File transfers from the Laboratory Information System (LIMS) for HPV screening results, and vaccination status extracted from medical charts and the physical informed consent documents for this baseline report.
Outcomes	The main objective of the trial: The study aims to evaluate whether organised, concomitant HPV vaccination and HPV screening offered by the Swedish cervical screening program to all resident women aged 23-25 will result in a more rapid elimination of HPV infection in Sweden. This objective will be examined at the population level.<br>Secondary objectives of the trial:<br>The study will evaluate whether the addition of concomitant vaccination to the cervical screening program results in an improved efficiency and/or safety of the cervical screening program:<br>1) Protection of Gardasil 9 against HPV infection and against CIN2+ by Gardasil 9 HPV vaccine types. The effectiveness of one-dose vaccination, and to determine the effect of 2-dose vaccinations.<br>2) Efficiency will be measured by the yield of histopathologically confirmed high-grade cervical cancer precursors or cancer (cervical intraepithelial neoplasia grade 2, 3, or cervical cancer) in relation to the consumption of resources and convenience for the women.<br>3) Safety will be measured by evaluating the occurrence of obstetrical complications such as preterm births as well as by measuring the number of excised cervical specimens found to be histopathologically benign.

Plants

Seed stocks	NA as stated above
Novel plant genotypes	NA as stated above
Authentication	NA as stated above
